# Estimating the impact of antiretroviral treatment on adult mortality trends in South Africa: A mathematical modelling study

**DOI:** 10.1371/journal.pmed.1002468

**Published:** 2017-12-12

**Authors:** Leigh F. Johnson, Margaret T. May, Rob E. Dorrington, Morna Cornell, Andrew Boulle, Matthias Egger, Mary-Ann Davies

**Affiliations:** 1 Centre for Infectious Disease Epidemiology and Research, University of Cape Town, Cape Town, South Africa; 2 School of Social and Community Medicine, University of Bristol, Bristol, United Kingdom; 3 Centre for Actuarial Research, University of Cape Town, Cape Town, South Africa; 4 Institute of Social and Preventive Medicine, University of Bern, Bern, Switzerland; Centers for Disease Control and Prevention, UNITED STATES

## Abstract

**Background:**

Substantial reductions in adult mortality have been observed in South Africa since the mid-2000s, but there has been no formal evaluation of how much of this decline is attributable to the scale-up of antiretroviral treatment (ART), as previous models have not been calibrated to vital registration data. We developed a deterministic mathematical model to simulate the mortality trends that would have been expected in the absence of ART, and with earlier introduction of ART.

**Methods and findings:**

Model estimates of mortality rates in ART patients were obtained from the International Epidemiology Databases to Evaluate AIDS–Southern Africa (IeDEA-SA) collaboration. The model was calibrated to HIV prevalence data (1997–2013) and mortality data from the South African vital registration system (1997–2014), using a Bayesian approach. In the 1985–2014 period, 2.70 million adult HIV-related deaths occurred in South Africa. Adult HIV deaths peaked at 231,000 per annum in 2006 and declined to 95,000 in 2014, a reduction of 74.7% (95% CI: 73.3%–76.1%) compared to the scenario without ART. However, HIV mortality in 2014 was estimated to be 69% (95% CI: 46%–97%) higher in 2014 (161,000) if the model was calibrated only to HIV prevalence data. In the 2000–2014 period, the South African ART programme is estimated to have reduced the cumulative number of HIV deaths in adults by 1.72 million (95% CI: 1.58 million–1.84 million) and to have saved 6.15 million life years in adults (95% CI: 5.52 million–6.69 million). This compares with a potential saving of 8.80 million (95% CI: 7.90 million–9.59 million) life years that might have been achieved if South Africa had moved swiftly to implement WHO guidelines (2004–2013) and had achieved high levels of ART uptake in HIV-diagnosed individuals from 2004 onwards. The model is limited by its reliance on all-cause mortality data, given the lack of reliable cause-of-death reporting, and also does not allow for changes over time in tuberculosis control programmes and ART effectiveness.

**Conclusions:**

ART has had a dramatic impact on adult mortality in South Africa, but delays in the rollout of ART, especially in the early stages of the ART programme, have contributed to substantial loss of life. This is the first study to our knowledge to calibrate a model of ART impact to population-level recorded death data in Africa; models that are not calibrated to population-level death data may overestimate HIV-related mortality.

## Abstract 2

Leigh Johnson and colleagues model the HIV epidemic in South Africa, using national level health statistics as inputs, to estimate the number of deaths avoided and extra years of life gained due to antiretroviral programs.

## Author summary

**Why was this study done?:**

- Several studies have shown that adult mortality levels in South Africa and other African countries have declined substantially since antiretroviral treatment (ART) became widely available. However, it is not clear how much of the decline in mortality is attributable to ART.
- The ART programmes in many African countries are massive public health investments, and it is important to quantify the return on these investments, for both local and international funders, in terms of life years saved.
- Previous models of HIV in Africa have been calibrated mostly to HIV prevalence data, and it is not known how inclusion of recorded death data in the calibration process could affect model estimates.

**What did the researchers do and find?:**

- We developed a mathematical model to simulate the HIV epidemic in South Africa, using as inputs South African demographic statistics, reported numbers of adults receiving ART, and levels of mortality in a sample of South African ART programmes.
- The model estimated that HIV caused 2.7 million deaths in South African adults between 1985 and 2014, but model estimates of HIV mortality increased substantially if the model was not calibrated using recorded death statistics.
- The model estimated that, compared to what would have happened if there had been no ART programme in South Africa, there were 1.72 million fewer HIV-related deaths in adults over the 2000–2014 period, equivalent to 6.15 million extra years of life lived as a result of the ART programme.
- The model also estimated that if there had been a quicker expansion of the South African ART programme, with more rapid adoption of World Health Organization (WHO) guidelines, 8.80 million life years would have been saved up to 2014.

**What do these findings mean?:**

- ART has had a dramatic impact on adult mortality in South Africa, but models that are not calibrated to population-level recorded death statistics may overestimate HIV mortality.
- The difference between the 6.15 million life years that were actually saved and the 8.80 million that could have been saved represents a tragic loss of life, which arose because the early rollout of ART was hindered by the Mbeki government, and because of other delays in the adoption of WHO guidelines.

## Introduction

Substantial declines in adult all-cause mortality have been observed in South Africa since the mid-2000s [1–4], consistent with trends in other African countries [5–8]. Although these reductions are commonly attributed to the impact of antiretroviral treatment (ART) on HIV-related mortality, there has been no formal assessment of the extent to which the observed reduction in mortality is explained by the introduction of ART. A number of other factors could partly explain the reductions in mortality. First, adult HIV incidence in South Africa and other African countries peaked in the 1990s and has since declined substantially [9–11]. Some of the reduction in mortality may thus be due to the stage of the HIV epidemic. Second, HIV may be evolving towards a less virulent form [12,13]. Third, reductions in all-cause mortality could be a reflection of reductions in non-HIV mortality. By fitting mathematical models to mortality and HIV prevalence data, it is possible to evaluate which factors best explain the observed reductions in adult mortality.

Most modelling studies have relied on data from ART cohorts established for research purposes, such as those participating in the International Epidemiology Databases to Evaluate AIDS (IeDEA) collaboration [14]. However, estimation of mortality from these cohorts is challenging, and few modelling studies have considered the biases that may arise. Mortality rates are highly heterogeneous between cohorts [15,16], and there is concern that research cohorts may not be representative of routine ART services in developing countries. There is also concern that average follow-up times in ART cohorts are typically short, such that mortality estimates are biased towards the high mortality observed at short ART durations [17]. Even when mathematical models allow for differences in mortality by ART duration, they typically do so using a piecewise-constant assumption [18,19], which leaves potential for exaggeration of mortality at longer ART durations. Finally, many patients who die while on ART are not recorded as having died and are instead classified as lost to follow-up [20], leading to underestimation of mortality.

South Africa is an ideal setting in which to evaluate the impact of ART on mortality at a population level, as it has one of the highest rates of vital registration in sub-Saharan Africa [21], with around 94% of all adult deaths recorded in recent years [22–25]. It also has well-established ART research cohorts, many of which participate in the IeDEA–Southern Africa (IeDEA-SA) collaboration [26]. By linking patient records to the South African vital registration system, researchers have been able to identify an estimated 96.2% of all adult deaths in patients with ID numbers [22]. South Africa’s public sector ART programme began in 2004, and although the early rollout of ART was hindered by the Mbeki government [27], ART access subsequently improved dramatically [28], with the South African ART programme becoming the biggest in the world.

This study aims to evaluate how much of the reduction in adult mortality in South Africa is attributable to the impact of ART, using a mathematical modelling approach. As most previous mathematical models of HIV in Africa have been calibrated only to HIV prevalence data, this study also aims to assess how sensitive model estimates of HIV mortality are to the inclusion of mortality data in the calibration process.

## Methods

The Thembisa model was used to simulate HIV transmission, disease progression, and mortality in South Africa. The model has previously been described [19], and S1 Text provides a more detailed explanation of the model parameters most relevant to the present analysis; the model is also freely available online (https://www.thembisa.org, Version 3.2 downloads). Briefly, the model is a deterministic model that divides the population into age- and sex-specific cohorts and ‘risk groups’ that are defined in terms of marital status and propensity for concurrent partnerships. Non-HIV mortality assumptions are based on an analysis of South African cause-of-death statistics [2]. The simulation of the HIV epidemic starts in 1985, based on an assumed initial prevalence of HIV in women in the high risk group. HIV incidence is simulated based on assumptions about rates of partnership formation, commercial sex activity, marriage, coital frequency, and condom use (all of which vary in relation to age and sex), as well as assumed probabilities of transmission per sex act. Probabilities of HIV transmission per act of unprotected sex depend on sex, relationship type, and the HIV stage of the HIV-positive partner. Transmission probabilities are assumed to be reduced after ART initiation, depending on assumed rates of viral suppression, and the female-to-male transmission probability is also assumed to be reduced if the male partner is circumcised.

### Progression of HIV disease

After acquiring HIV, adults (ages 15 years and older) are assumed to progress through 5 stages of HIV infection in the absence of ART (acute infection, CD4 ≥ 500 cells/μl, CD4 350–499 cells/μl, CD4 200–349 cells/μl, and CD4 < 200 cells/μl). Rates of progression between CD4 stages and untreated HIV mortality rates depend on the individual’s age and sex. Due to uncertainty regarding the average untreated HIV survival time and the effects of age and sex on HIV survival, prior distributions are assigned to represent the uncertainty around these parameters (Table 1). The model allows for a possible change in HIV virulence over time; a prior distribution represents the uncertainty around parameter *E*, the relative rate of progression between CD4 stages for each additional calendar year (Table 1). In year *t*, the rate of progression from one CD4 stage to the next is proportional to *E*^*t* − 1999^. Any change in virulence is likely to affect set point viral loads (SPVLs) [29–31], which in turn affect transmission probabilities [32–34]. The model therefore allows for a change over time in HIV transmission probabilities, with the transmission probability per sex act in year *t* being proportional to *E*^αϕ(*t* − 1999)^, where α is the ratio of the increase in infectivity (on a log scale) to the increase in disease progression (on a log scale) for each unit change in SPVL (Table 1), and ϕ is a constant (2.5).

**Table 1:** Comparison of prior and posterior distributions.

Parameter	Prior, mean (95% CI)	Posterior, mean (95% CI)	Prior source
*Untreated HIV mortality parameters*			
Average male survival in absence of ART (years)	12.00 (10.12–14.04)	11.48 (11.17–11.82)	[35–37]
RR of HIV disease progression in women	0.960 (0.864–1.060)	0.921 (0.896–0.948)	[35,38]
Increase in HIV disease progression per 10-year increase in age	0.180 (0.082–0.315)	0.235 (0.209–0.259)	[38–41]
RR of HIV disease progression per calendar year (E)	1.000 (0.987–1.013)	0.978 (0.972–0.983)	[12,13,36,42–44]
*Treated HIV mortality parameters*			
IeDEA-SA bias in first 6 months of ART	1.850 (1.004–2.951)	1.368 (1.068–1.708)	—
Ratio of IeDEA-SA bias >42 months after ART start to bias <6 months after ART start	0.800 (0.616–1.008)	0.619 (0.525–0.706)	—
Reduction in mortality* per unit increase in ART initiation rate (at CD4 < 200 cells/μl) over last 3 years	7.50 (2.29–15.76)	4.76 (3.71–5.75)	[45,46]
*HIV transmission parameters*			
Ratio of increase in infectivity* to increase in disease progression*, per unit change in SPVL	1.000 (0.376–1.923)	0.111 (0.095–0.128)	[29–34]
Initial HIV prevalence for women in high risk group	0.100% (0.005%–0.195%)	0.193% (0.184%–0.198%)	[47]
Male-to-female transmission probability in short-term relationships, per sex act	0.0120 (0.0043–0.0236)	0.0198 (0.0188–0.0206)	[48,49]
Female-to-male transmission probability in short-term relationships, per sex act	0.0080 (0.0032–0.0149)	0.0075 (0.0072–0.0079)	[50,51]

*On a natural log scale.

IeDEA-SA, International Epidemiology Databases to Evaluate AIDS–Southern Africa; RR, relative rate; SPVL, set point viral load.

The model simulates HIV testing and diagnosis and assumes that once individuals have been diagnosed, they can start ART immediately (if eligible) or defer ART. The model allows for changes over time in South African ART eligibility criteria, and allows for differences in ART initiation rates between ART-eligible diagnosed adults depending on sex and CD4 stage. The model has been calibrated to match the reported numbers of adults on ART in the public and private sectors [28].

Once individuals have started ART, their HIV-related mortality rates depend on their age, sex, CD4 stage at ART initiation, and time since ART initiation. These rates are based on relative survival models fitted to IeDEA-SA data; the models estimate HIV-specific mortality in excess of background (non-HIV) mortality after linking patient records to the national death registry [52]. To allow for the possibility that the IeDEA-SA estimates may be biased, 2 multiplicative adjustment factors are specified: one for the first 6 months after ART initiation and one for ART durations of over 42 months (adjustments at other durations are interpolated from these). Prior distributions are specified to represent the uncertainty around the first adjustment and the ratio of the second to the first (Table 1). The model allows the mortality rates in untreated individuals with CD4 < 200 cells/μl and treated individuals with baseline CD4 < 200 cells/μl to depend on the average rate of ART initiation over the last 3 years (since high rates of ART initiation mean that most patients starting ART with CD4 < 200 cells/μl will start ART soon after crossing the 200-cells/μl threshold, leaving relatively few untreated individuals at very low CD4 counts).

### Calibration and validation

A Bayesian approach was adopted in fitting the model to South African HIV prevalence and mortality data. Although most of the HIV transmission and sexual behaviour parameters were fixed at the posterior means estimated when the model was previously fitted to HIV prevalence data [19], prior distributions were specified to represent the uncertainty in the 4 transmission parameters given in Table 1. Prior distributions were also specified for the 7 disease progression and mortality parameters given in Table 1. A likelihood function was defined to represent the model fit to the all-cause recorded death statistics over the 1997–2014 period [1], disaggregated by sex and 5-year age group, and adjusted for incomplete registration [22] and lower completeness in the period prior to 2004 [53]. Only the deaths in the 20–59-year age range were included for calibration purposes, as HIV contributes relatively little to mortality at older ages. The likelihood function also represents the model fit to age-specific HIV prevalence data from national antenatal clinic surveys (1997–2013) and household surveys (in 2005, 2008, and 2012) [54]. Posterior estimates of the model parameters that provide the best fit to the HIV prevalence and recorded death data were simulated using incremental mixture importance sampling [55]. Further technical details are provided in S1 Text. Posterior model estimates were validated by comparing the model estimates of the proportions of HIV-positive adults in different CD4 categories to survey estimates of the corresponding proportions. Model results were also compared to those obtained when only the HIV prevalence data were used in the likelihood function (i.e., the approach that has traditionally been used in the calibration of HIV models).

### Counterfactual scenarios

The model simulations were compared with simulations in 2 counterfactual scenarios: a worst-case scenario that considers what would have happened in the absence of any ART and a best-case scenario that considers what would have happened if South Africa had followed WHO guidelines on ART eligibility immediately upon publication [56–60], with diagnosed patients starting ART an average of 6 months after reaching ART eligibility [61]. Adult life years saved by ART were estimated by subtracting the adult population size (individuals aged 15 years and older) in the worst-case scenario from that in the main scenario, and summing the differences over the period from 1 July 2000 to 30 June 2014. Similarly, potential life years gained in the best-case scenario were estimated by summing the differences in adult population size between the best- and worst-case scenarios.

## Results

### Model calibration

**Fig 1:**
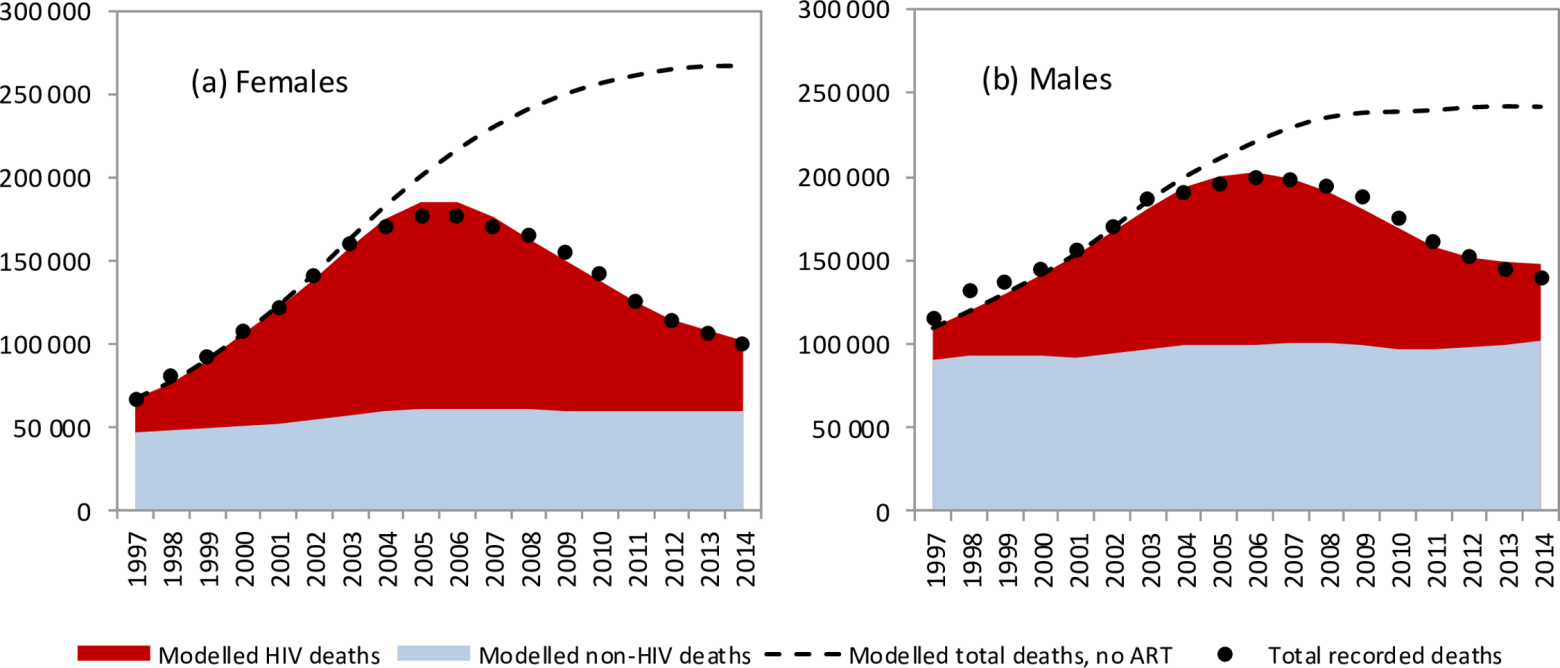
Trends in modelled and recorded deaths in adults (ages 20–59 years). Modelled and recorded HIV deaths in (a) females and (b) males for the years 1990–2014. Shaded areas represent posterior means from the main analysis, and dashed lines represent model estimates from the ‘no ART’ counterfactual. Dots represent recorded death estimates, after adjustment for incomplete vital registration.

The model was in close agreement with recorded levels of adult mortality in South Africa, which rose rapidly to peak in 2006 before declining (Fig 1). Similar consistency was noted when the model was compared with recorded death statistics over each 5-year age range (Fig D in S1 Text). The model was also consistent with age- and sex-specific HIV prevalence levels in South African national surveys (Figs E and F in S1 Text). Model validations were performed against surveys of CD4 distributions in HIV-positive South African populations, and model estimates were found to be in reasonable agreement with survey data (Fig J in S1 Text). However, when the model was calibrated only to HIV prevalence data, model estimates of mortality substantially exceeded observed mortality after 2003 (Fig L in S1 Text).

### Posterior estimates of model parameters

Posteriors estimates of model parameters were in most cases consistent with prior distributions (Table 1). However, there was evidence of a change towards lower HIV virulence over time (relative rate of disease progression of 0.978 [95% CI: 0.972–0.983] per calendar year). The bias in the IeDEA-SA estimates of mortality differed by time since ART initiation: during the first 6 months after ART start, mortality rates in the model were estimated to be 1.37 (95% CI: 1.07–1.71) times those estimated from IeDEA-SA cohorts, but at >42 months after ART initiation, mortality rates in the model were estimated to be lower than those estimated in IeDEA-SA cohorts.

### Mortality attributable to HIV/AIDS

**Fig 2:**
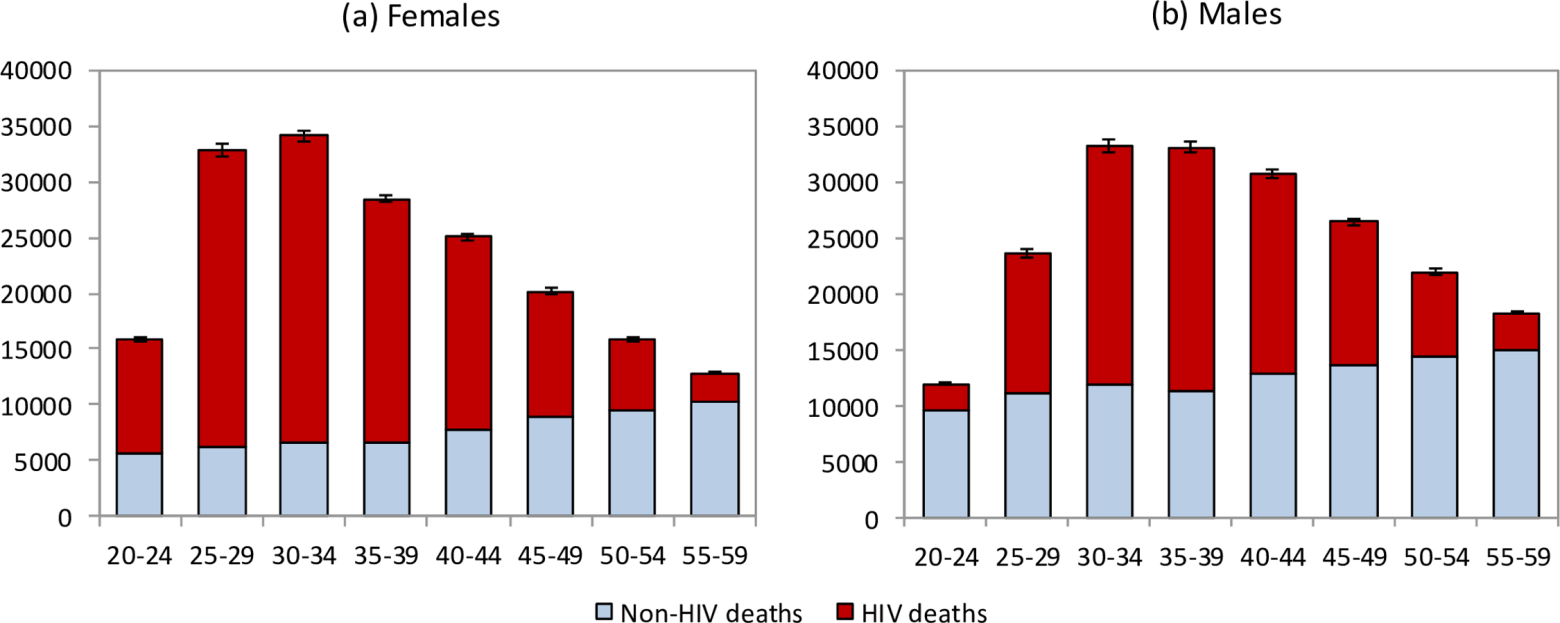
Age and sex differences in mortality levels (1 July 2005–30 June 2006). HIV and non-HIV deaths by 5-year age group in (a) females and (b) males. Bars represent posterior means from the main analysis. Error bars represent 95% confidence intervals around model estimates of total deaths (HIV and non-HIV combined).

The model estimates that in the 1985–2014 period, 2.70 million adult HIV-related deaths occurred in South Africa (95% CI: 2.66 million–2.75 million). Adult HIV deaths are estimated to have peaked at 231,000 per annum in 2006 (95% CI: 227,000–235,000) and declined to 95,000 in 2014 (95% CI: 91,000–99,000). Although HIV/AIDS initially accounted for more deaths in women than in men, numbers of HIV/AIDS deaths declined to similar levels in men and women in 2014 (Fig 1). The age pattern of HIV/AIDS mortality over the 2005–2006 period (1 July 2005–30 June 2006) differed for men and women, with HIV-related deaths in women being highest in the 25–34-year age range, and those in men being highest in the 30–39-year age range (Fig 2). Non-HIV mortality rates were substantially lower in women than in men, with the result that HIV accounted for a greater fraction of deaths in women (up to 81% of deaths in women aged 25–29 years over the 2005–2006 period). The model estimated a 17% greater number of adult HIV-related deaths up to 2014 if it was calibrated only to HIV prevalence data (3.16 million, 95% CI: 2.83 million–3.55 million). The greatest divergence in HIV mortality estimates, compared to that in the main analysis, occurred in the more recent years: using only HIV prevalence data to calibrate the model yielded an estimate of 161,000 HIV deaths (95% CI: 138,000–186,000) in 2014, 69% (95% CI: 46%–97%) higher than when the model was calibrated to both HIV prevalence and mortality data.

**Fig 3:**
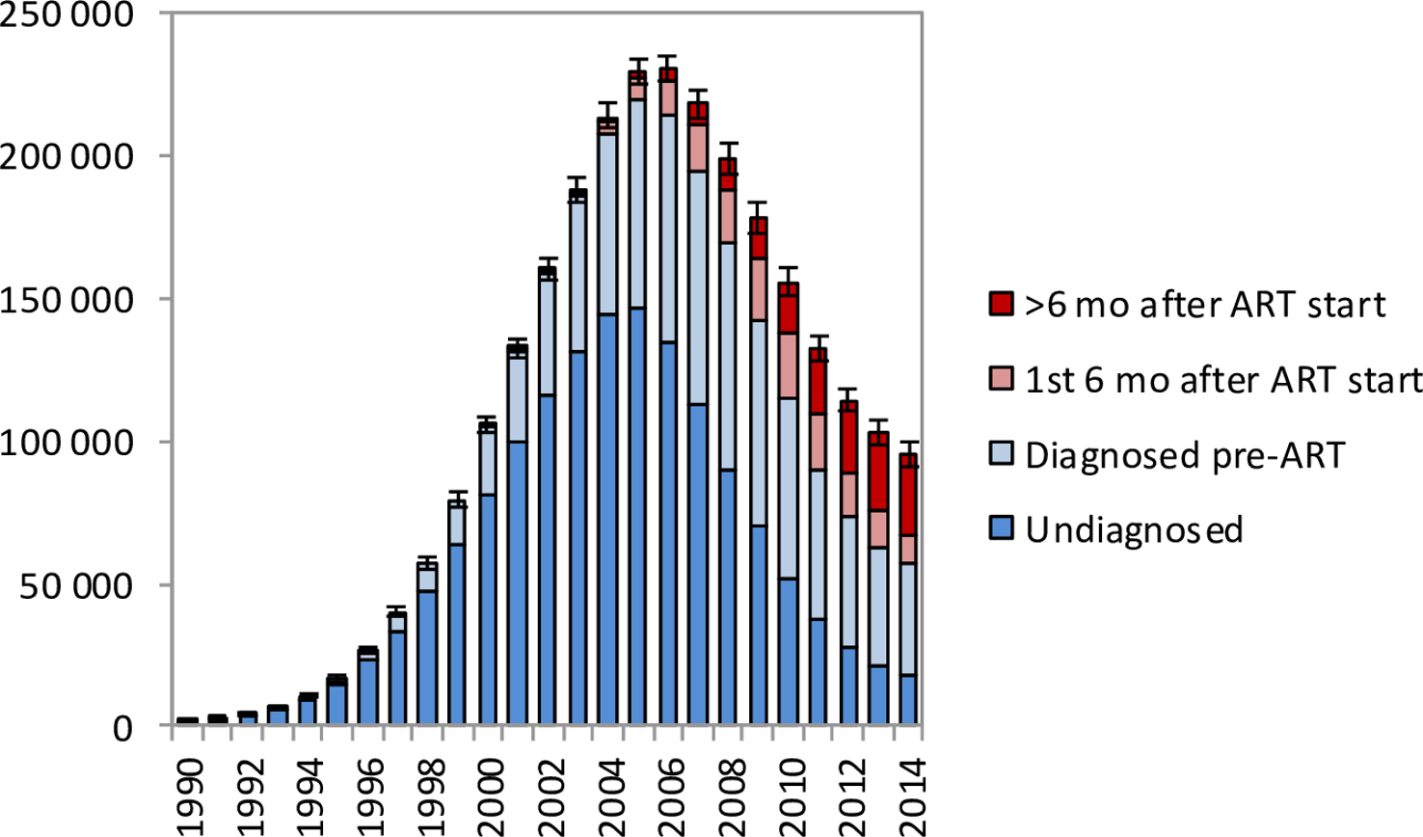
HIV-related deaths in adults for the years 1990–2014 by level of engagement in HIV care. Bars represent posterior means from the main analysis. Error bars represent 95% confidence intervals around model estimates of total HIV-related deaths in adults.

In the period up to 2005, most HIV-related deaths in South African adults occurred in individuals who were undiagnosed, but in recent years an increasingly high proportion of HIV-related deaths occurred in individuals who had been diagnosed but were ART-naïve (Fig 3). In 2014, the model estimates that 18.3% (95% CI: 17.2%–19.5%) of adult HIV-related deaths occurred in individuals who were undiagnosed, 41.7% (95% CI: 34.6%–48.0%) occurred in individuals who were diagnosed but ART-naïve, 10.2% (95% CI: 8.6%–11.9%) occurred in individuals within 6 months of starting ART, and 29.8% (95% CI: 24.6%–36.2%) occurred in individuals who had started ART more than 6 months prior to death.

### Life years saved by ART

**Fig 4:**
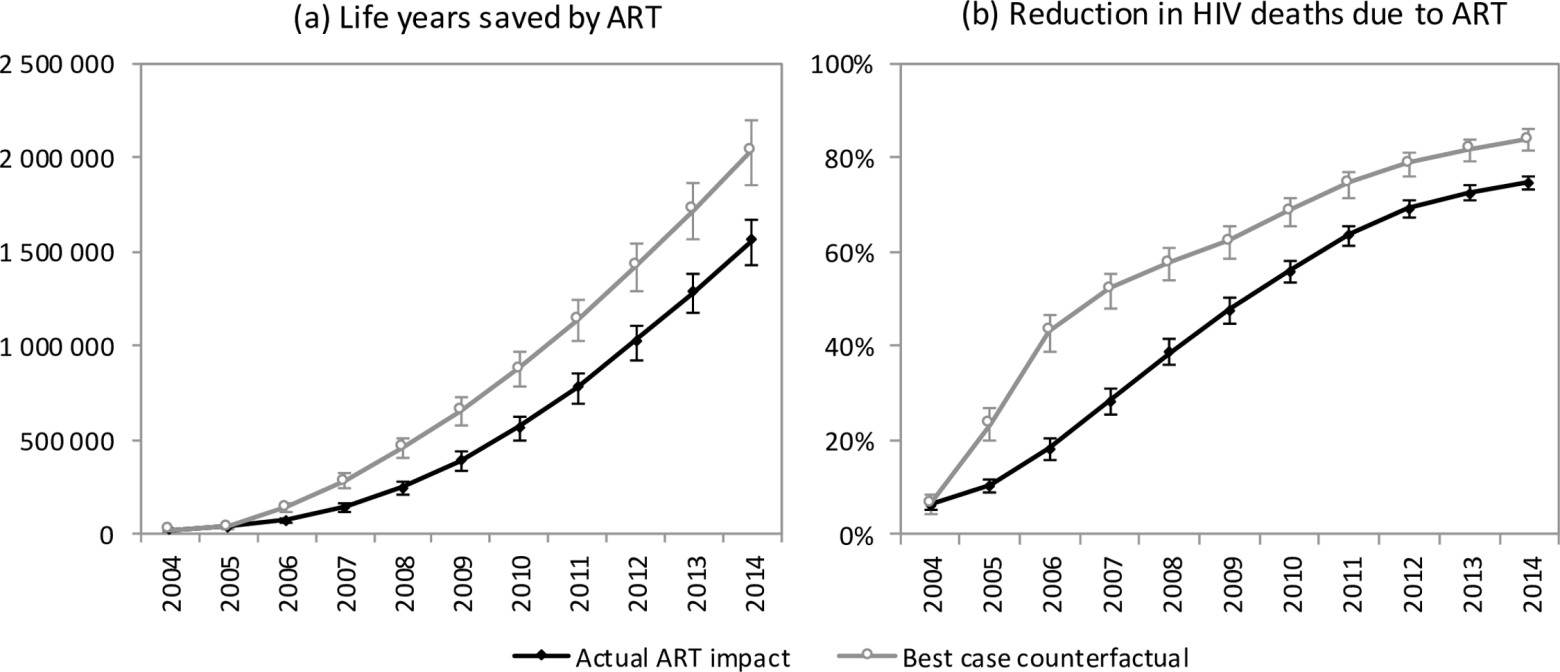
Impact of ART on mortality. Life years saved by ART (a) and reduction in HIV deaths due to ART (b) for the years 2004–2014. Error bars represent 95% confidence intervals around model outputs. Savings are calculated relative to a ‘no ART’ counterfactual. In the ‘actual ART impact’ scenario, actual rates of ART enrolment in South Africa are entered into the model, and the model results are compared to the ‘no ART’ counterfactual. In the ‘best-case counterfactual’, the rates of ART enrolment entered into the model are those that would have been expected if South Africa had adopted WHO guidelines immediately upon publication and promoted ART rollout more aggressively.

The model estimates that in the absence of ART, adult HIV-related deaths would have continued to increase over time, reaching 376,000 per annum in 2014 (Fig 1). In the 2000–2014 period, the South African ART programme is estimated to have saved 6.15 million life years in adults (95% CI: 5.52 million–6.69 million), and the cumulative number of adult HIV-related deaths was reduced by 1.72 million (95% CI: 1.58 million–1.84 million) relative to what would have been expected in the absence of ART. This compares with a potential saving of 8.80 million (95% CI: 7.90 million–9.59 million) life years and 2.20 million (95% CI: 2.00 million–2.37 million) fewer HIV-related deaths if South Africa had moved swiftly to implement WHO guidelines and had achieved high levels of ART uptake in HIV-diagnosed individuals (Fig 4A). A higher number of life years saved (6.71 million, 95% CI: 5.56 million–8.02 million) and HIV deaths averted (1.85 million, 95% CI: 1.64 million–2.07 million), relative to the ‘no ART’ counterfactual, was also estimated when the model was fitted only to HIV prevalence data. The annual percentage reduction in HIV-related mortality due to ART increased steadily after the start of the public sector ART rollout in 2004, reaching 74.7% (95% CI: 73.3%–76.1%) in 2014 (Fig 4B). However, a much more dramatic reduction in HIV-related mortality could have been achieved in the best-case scenario, if ART rollout had been more timely.

## Discussion

HIV/AIDS has had a profound impact on adult mortality in South Africa, causing 2.7 million deaths in the 1985–2014 period. It is encouraging to see the dramatic impact that ART has had, saving 6.2 million adult life years in the period up to 2014, and reducing AIDS mortality in adults to only a quarter of what it would have been in the absence of ART. However, the slow pace of ART rollout, against a backdrop of political resistance to ART in the early 2000s [27], has had tragic consequences: our model estimates that the saving in life years could have been 2.7 million greater if the South African government had promoted ART more aggressively and adopted WHO guidelines in a more timely manner. These results are based on a model that has been calibrated to detailed and extensive HIV prevalence data, mortality data, HIV testing data, ART programme data, and estimates of mortality rates in ART research cohorts. Our model estimates that over 40% of adult HIV-related deaths in 2014 occurred in individuals who had been diagnosed but had not started ART. Our model also suggests that the observed mortality decline in South Africa over the last decade cannot be attributed only to the impact of ART, and changes in HIV virulence could also be partly responsible for the reduction.

Our estimates of AIDS mortality in South Africa are similar to those obtained in the Second National Burden of Disease Study (Fig N in S1 Text), which analysed cause-of-death data in South Africa, recoding deaths that did not appear to have been correctly recorded in the South African vital registration system [2]. However, our estimates of AIDS mortality levels are substantially lower than those published by the Joint United Nations Programme on HIV/AIDS (UNAIDS) and the Institute for Health Metrics and Evaluation [62] (Fig N in S1 Text). Similar lack of agreement between model estimates of mortality in South Africa and recorded levels of mortality have been noted in a previous comparison of 10 different HIV models [63], and similar inconsistencies between WHO estimates and estimates from Demographic Surveillance System sites have been observed in Uganda [8]. The vast majority of these models have not been calibrated to mortality data, and the lack of agreement with recorded death statistics is thus not surprising. Indeed, our model also overestimated recorded levels of adult mortality when it was calibrated only to HIV prevalence data, with the estimated levels of AIDS mortality in 2014 in this analysis being about 69% greater than in the main analysis. There have been few prior attempts to fit HIV models to recorded death statistics in the countries most heavily affected by HIV, and those models that have done so limited their focus to mortality in the pre-ART era [9,64,65]. It is important that efforts are made to integrate mortality data into HIV model calibration procedures if these models are to reflect accurately the impact of ART.

A number of factors could explain why other models have tended to overestimate HIV mortality in South Africa. Most importantly, few previous models have considered the possibility of changing HIV virulence. Our results suggest that HIV may be evolving towards a less virulent form, consistent with evidence from other African studies [12,13]. These changes may be the result of a trade-off between virulence and transmission potential [13,66], or they may be the result of the fitness costs associated with HIV mutating to escape cytotoxic T lymphocyte responses [12,67].

Most mathematical models of ART impact are parameterized using mortality data from ART research cohorts, but this analysis suggests that these data are biased in complex ways. South African cohorts participating in the IeDEA-SA collaboration are predominately urban, and many of the associated programmes operate with strong support from non-governmental organisations and academic institutions, making them untypical of the general public health sector. The higher socioeconomic status of patients in urban cohorts and the higher staff-to-patient ratios in IeDEA-SA cohorts might be expected to contribute to lower mortality rates in the IeDEA-SA cohorts compared to the general public health sector [68–70]. However, this bias may become less significant at longer durations after ART initiation because patients frequently move between services or interrupt ART [71,72], while the vital registration system continues to record deaths after patients have left their original IeDEA-SA treatment cohorts. In addition, the bias may be offset by a counteracting bias due to the assumption of piecewise-constant mortality over different treatment durations.

A limitation of this analysis is that we have calibrated the Thembisa model to all-cause mortality data rather than HIV-related mortality data, without making any allowance for uncertainty in non-HIV mortality rates. Cause of death is poorly recorded in the South African vital registration system [73], with HIV-related causes being particularly susceptible to misclassification [74]. It is thus not possible to derive reliable estimates of HIV-related deaths directly from the vital registration data unless assumptions are made about patterns of misreporting, as in the Second National Burden of Disease Study [2]. The non-HIV mortality assumptions in the Thembisa model are derived from the Second National Burden of Disease Study, and the cause-of-death data are therefore to some extent already implicit in the model. Non-HIV mortality rates over the 20–59-year age range are assumed to have declined by 15%–34% in men and by 7%–20% in women over the 1995–2014 period; assuming a more modest decline in non-HIV mortality would lead to a smaller estimated impact of HIV on South African mortality.

Another limitation of this analysis is that we have not allowed for changes in ART effectiveness over time, such as might be expected following the introduction of tenofovir and fixed dose combinations. However, a recent analysis of IeDEA-SA data suggests that there has been little change in ART mortality rates over time, after controlling for changes in baseline CD4 count and changes in ART duration [75]. Some bias could also be introduced by the assumption of a constant annual change in HIV virulence: other analyses suggest that the change in virulence might be more rapid in the early stages of the epidemic, and the trend might not be consistently in the same direction [44,76,77]. More sophisticated modelling is required to identify the likely mechanisms behind the putative virulence changes. It is also worth noting that we have not considered uncertainty in several drivers of HIV incidence, and it is thus not possible to draw conclusions about the effect of changes in virulence on trends in HIV incidence.

This study does not consider the macroeconomic consequences of the ART programme in South Africa. It is likely that increased labour productivity and reduced costs of orphan care have contributed positively to South Africa’s gross domestic product [78], and one study estimated that output per capita could increase by 12% in South Africa if all HIV-positive individuals received ART [79]. However, a recent review found little consistency between studies that quantified the macroeconomic impact of HIV [80]. Further macroeconomic modelling is required to estimate the economic benefits of the ART-related savings in life years. This study also does not consider the impact of ART on South Africa’s tuberculosis (TB) epidemic, which has been shown to be substantial in other modelling studies [81]. Independently of the ART programme, there have been several other TB interventions that may have reduced TB mortality in HIV-positive individuals, such as isoniazid preventive therapy for HIV-positive individuals, improved TB diagnosis, TB case finding in HIV-positive individuals, and improvements in TB treatment [82]. Although we have attributed much of the improvement in untreated HIV-positive mortality in South Africa to changes in HIV virulence, it is possible that some of the improvement was in fact due to these advances in TB care and prevention.

This analysis demonstrates that the reductions in adult mortality that have been observed in South Africa are not attributable to the stage of the HIV epidemic. In fact, AIDS mortality rates would have continued to rise in the absence of ART (Fig 1), and a simple comparison of mortality rates in recent years to those in 2006 therefore does not do justice to the extent of the mortality reduction achieved by ART. The reductions in adult mortality are also not attributable to improvements in non-HIV mortality, as declines in non-HIV mortality rates since 1997 have been relatively modest [4,83].

Although South Africa has recently adopted a policy of universal ART eligibility, many challenges remain in reducing AIDS mortality. The high proportion of HIV-related deaths occurring in individuals who have been diagnosed but have not started ART suggests that many individuals who know they are HIV-positive and are ART-eligible are either reluctant or unable to start ART. This is consistent with the experience of a recent trial of treatment-as-prevention in rural South Africa, in which only 47% of newly diagnosed adults linked to care within 6 months of diagnosis [84], and estimates from the same community show that most HIV deaths occur pre-ART [85]. In order for ART to be more effective, both in reducing AIDS mortality and in preventing HIV transmission, it will be critical to ensure more timely ART initiation after HIV diagnosis. Simplified models of ART initiation are needed in order to achieve greater uptake of ART following diagnosis, for example, same-day ART initiation or expedited ART initiation [86,87]. In addition, education programmes may be required in order to raise awareness of the benefits of early ART initiation, and to address misconceptions about ART [88]. Our model also suggests that an increasingly high proportion of HIV-related deaths (30% in 2014) occur more than 6 months after ART initiation. Interventions to improve ART retention and increase viral suppression will therefore also be important in reducing future levels of AIDS mortality in South Africa.

## Supporting information

S1 Text Supplementary material. (DOC)

## Abbreviations

ART: antiretroviral treatment
IeDEA-SA: International Epidemiology Databases to Evaluate AIDS–Southern Africa
SPVL: set point viral load
TB: tuberculosis
