# Supplementary material for: Estimating the impact of antiretroviral treatment on adult mortality trends in South Africa: A mathematical modelling study
Source: PLoS Med. 2017 Dec 12;14(12):e1002468. doi: 10.1371/journal.pmed.1002468 (PMC5726614; doi:10.1371/journal.pmed.1002468)
Supplement: S1 Text — (DOC) [file pmed.1002468.s001.doc]

Text S1: Supplementary material

Table of Contents

[1. Model of HIV survival 3](#__RefHeading___Toc487366936)

[1.1 Overview of HIV survival model 3](#__RefHeading___Toc487366937)

[1.2 Mortality in untreated HIV-positive adults 3](#__RefHeading___Toc487366938)

[1.2.1 Average HIV survival time in the absence of ART 4](#__RefHeading___Toc487366939)

[1.2.2 Effect of sex on HIV disease progression and mortality ($\varpi$) 5](#__RefHeading___Toc487366940)

[1.2.3 Effect of age on HIV disease progression and mortality (k) 6](#__RefHeading___Toc487366941)

[1.2.4 Extent of HIV viral evolution (E) 7](#__RefHeading___Toc487366942)

[1.3 Mortality in adults on ART 8](#__RefHeading___Toc487366943)

[1.3.1 Baseline mortality rates (D_g,d,c_) 8](#__RefHeading___Toc487366944)

[1.3.2 Age adjustment factor (ψ_g_) 9](#__RefHeading___Toc487366945)

[1.3.3 IeDEA bias adjustment factor (I_d_) 10](#__RefHeading___Toc487366946)

[1.3.4 Time-dependent adjustment factors (B(t) and C(t)) 13](#__RefHeading___Toc487366947)

[1.4 Modelling of HIV diagnosis and ART initiation 14](#__RefHeading___Toc487366948)

[2. Model of HIV transmission 16](#__RefHeading___Toc487366949)

[3. Model fitting procedure 18](#__RefHeading___Toc487366950)

[3.1 Prior distributions 18](#__RefHeading___Toc487366951)

[3.2 Likelihood function 19](#__RefHeading___Toc487366952)

[3.2.1 Recorded death data 19](#__RefHeading___Toc487366953)

[3.2.2 Antenatal HIV prevalence data 21](#__RefHeading___Toc487366954)

[3.2.3 Household survey HIV prevalence data 23](#__RefHeading___Toc487366955)

[3.3 Posterior simulation 23](#__RefHeading___Toc487366956)

[4. Additional results 25](#__RefHeading___Toc487366957)

[4.1 Model calibration and validation in main and alternative analyses 25](#__RefHeading___Toc487366958)

[4.2 Comparison of model results when fitting only to prevalence data and only to mortality data 35](#__RefHeading___Toc487366959)

[4.3 Comparison with recently-published AIDS mortality estimates from other models 38](#__RefHeading___Toc487366960)

[References 39](#__RefHeading___Toc487366961)

[Appendix A: Modelling of changes in mortality at low CD4 counts 47](#__RefHeading___Toc487366962)

# 1. Model of HIV survival

## 1.1 Overview of HIV survival model

The model of HIV survival in adults is summarized in Fig A. Individuals who acquire HIV are assumed to enter an ‘acute HIV’ state. After having left the acute HIV state, HIV-positive adults are stratified according to their current CD4 count and knowledge of their HIV status/HIV testing history. All infected adults are assumed to have initial CD4 counts above 500 (based on the CD4 distributions observed in HIV-negative South Africans ), which decline over the course of HIV infection. After having started ART, individuals are stratified according to their baseline CD4 category and time since ART initiation. The sections that follow describe the assumptions made in parameterizing this model.

Fig A: Multi-state model of survival in HIV-positive adults

HIV mortality from all untreated states with CD4 <350 cells/μl and all treated states is included (not shown).

## 1.2 Mortality in untreated HIV-positive adults

In untreated individuals, we define the symbol $\lambda_{g,s}(x,t)$ to be the annual rate of transition from HIV state *s* to state (*s* + 1) in untreated HIV-positive individuals of sex *g* (1 = males, 2 = females) who are aged *x*, in year *t*. This is calculated as

$\lambda_{g,s}(x,t)=\lambda_{s}\varpi^{g-1}\left(1+k\right)^{(x-30)/10}E^{t-1999}$, (1)

where *λ_s_* is the rate that applies in men aged 30 in 1999, $\varpi$ is the factor by which HIV disease progression is adjusted in women, *k* is the proportional increase in the rate of disease progression per 10-year increase in age, and *E* is factor by which the disease progression rate increases with each additional calendar year. Similarly, we define the symbol $\mu_{g,s}(x)$ to be the annual HIV-related mortality rate in HIV state *s* in untreated individuals of sex *g* who are aged *x*. This is calculated as

$\mu_{g,s}(x)=\mu_{s}\varpi^{g-1}\left(1+k\right)^{(x-30)/10}$, (2)

where *μ_s_* is the HIV mortality rate that applies in men aged 30. The adjustment factors for the effects of age and sex on HIV disease progression are thus the same as the adjustment factors for the corresponding effects on HIV-related mortality, but mortality rates are assumed not to depend on calendar year.

### 1.2.1 Average HIV survival time in the absence of ART

For the purpose of setting the average HIV survival time, we first consider a simplified model in which there is no dependency of disease progression and mortality on any of the covariates (age, sex or calendar year). As HIV-related mortality rates above CD4 350 cells/μl appear to be very low, we set $\mu_{1}=\mu_{2}=\mu_{3}=0$. The mean survival time in the absence of ART is

$\pi=\frac{1}{\lambda_{1}}+\frac{1}{\lambda_{2}}+\frac{1}{\lambda_{3}}+\frac{1}{\lambda_{4}+\mu_{4}}+\frac{\lambda_{4}}{\lambda_{4}+\mu_{4}}\times\frac{1}{\mu_{5}}$. (3)

In a previous modelling exercise , which involved fitting a South African HIV model to survey estimates of CD4 distributions in HIV-infected adults, the parameters $\lambda_{2}$, $\lambda_{3}$ and $\lambda_{4}$ were estimated to be 0.30, 0.48 and 0.32 respectively,[[1]](#footnote-2) although the $\lambda_{2}$ parameter was defined differently in that analysis as the model did not include an acute stage of HIV infection. If we use the symbol $\hat{\lambda}_{s}$ to represent the parameter values derived from these previous estimates and assume that the average duration of the acute HIV infection stage is 3 months , then $\hat{\lambda}_{1}=1/0.25$ and $\hat{\lambda}_{2}=1/\left(\left(1/0.30\right)-0.25\right)$. The mortality parameters in the same analysis, $\hat{\mu}_{4}$ and $\hat{\mu}_{5}$, were estimated at 0.027 and 0.21 respectively . Substituting these estimated values into equation (3) yields an estimated mean survival time of $\hat{\pi}$ = 11.72 years in the absence of ART.

Although these previous estimates are useful as a starting point, the model on which they are based was not calibrated to South African mortality data. As we wish to allow for uncertainty regarding the mean survival time in South Africa when fitting the model to recorded numbers of deaths in South Africa, we specify a prior distribution for the parameter $\pi$. Previous studies have estimated that for HIV-positive men in South Africa the median time from HIV infection to death, in the absence of ART, is 11.6 years , though this could be an under-estimate of $\pi$, as mean survival time usually exceeds median survival time. In a pooled analysis of African studies of untreated HIV survival, the sum of the average survival times before and after reaching different CD4 thresholds ranged between 13.7 and 14.6 years, depending on the CD4 threshold used . However, these estimates may be over-estimates, as patient follow-up time was censored at the point of starting ART, which can introduce a positive bias. To represent our uncertainty regarding the parameter $\pi$ we have therefore chosen a gamma prior distribution with a mean of 12 years and a standard deviation of 1 year. For a chosen $\pi$ value, we obtain the values $\lambda_{s}$ and $\mu_{s}$ using the equations

$\lambda_{s}=\hat{\lambda}_{s}\times\hat{\pi}/\pi$ (4)

and

$\mu_{s}=\hat{\mu}_{s}\times\hat{\pi}/\pi$. (5)

Our Bayesian approach therefore allows for uncertainty regarding the total untreated survival time, but assumes that the proportion of untreated survival spent in each CD4 category remains fixed at the levels estimated previously. This is appropriate because we are not attempting to recalibrate the model to CD4 data.

### 1.2.2 Effect of sex on HIV disease progression and mortality ($\varpi$)

Much research has been conducted to determine whether the course of untreated HIV infection is different in men and women. It is well-established that HIV-negative women have higher CD4 counts than HIV-negative men , which implies that women would be expected to have higher CD4 counts than men in the early stages of HIV infection. It is also well-established that women have lower viral loads than men in the early stages of HIV infection . These differences between men and women in early CD4 and viral load dynamics suggest longer HIV survival in women.

However, there is also some evidence to suggest that viral load increases more rapidly in women than in men, with viral loads in women ‘catching up’ with those in men by the time patients progress to advanced disease . In addition, some evidence suggests that after controlling for CD4 counts, AIDS mortality rates might be higher in women than in men . These factors could potentially offset the female survival advantage in early HIV infection.

Studies that have evaluated the overall survival time from HIV infection to death, in the absence of ART, have generally found no significant difference between men and women after controlling for age at HIV acquisition. A collaborative analysis of data from high income settings in the pre-ART era found that after controlling for age and other factors, there was a marginally lower mortality rate in women, which was not statistically significant (HR 0.89, 95% CI: 0.74-1.07) . Similarly, in a collaborative analysis of mortality data from developing countries, based on data collected prior to the availability of ART, the relative rate of death in women was less than one but not significantly so (HR 0.91, 95% CI: 0.71-1.16) .

Given the lack of clear evidence of an effect of sex on the overall HIV survival time in the absence of ART, we assign a gamma prior distribution to represent the uncertainty regarding the $\varpi$ parameter. This prior has a mean of 0.96 and a standard deviation of 0.05, which yields 2.5 and 97.5 percentiles of 0.86 and 1.06 respectively. If all the $\lambda_{s}$ and $\mu_{s}$ terms in equation (3) were multiplied by the lower limit of 0.86, this would imply an increase in the mean (*π*) by a factor of 1.16 (1/0.86). If one were to assume that overall HIV survival times were Weibull-distributed, with a fixed shape parameter of 2.5 , then the increase in the mean by a factor of 1.16 would imply a reduction in the Weibull scale parameter by a factor of 0.69 (1.16^-2.5^). Similarly, if the $\lambda_{s}$ and $\mu_{s}$ parameters in equation (3) were multiplied by the upper limit of 1.06, this would imply an increase in the Weibull scale parameter of 1.16. Since the Weibull scale parameter is directly proportional to the mortality rate, this range of Weibull scale parameter adjustments (0.69-1.16) is directly comparable to the hazard ratios cited in the previous paragraph. The 95% confidence intervals from the two collaborative analyses (0.74-1.07 and 0.71-1.16) are both within this range. Following the same logic, the prior mean of 0.96 is consistent with a hazard ratio of 0.90, which is similar to the estimates of 0.89 and 0.91 in the two collaborative studies.

### 1.2.3 Effect of age on HIV disease progression and mortality (*k*)

Several studies have shown that older age at seroconversion is associated with increased mortality rates. High estimates of the increase in mortality per 10-year increase in age have also been observed in rural Tanzania (HR 1.72, 95% CI: 1.34-2.59) , Canada (HR 1.38, 95% CI: 1.05-1.81) and Italy (HR 1.55, 95% CI: 1.31-1.83) . A pooled analysis of data from high-income countries found that the hazard ratio per 10-year increase in age was between 1.35 and 1.59, depending on the mode of HIV acquisition . This suggests average hazard ratios of around 1.5 per 10-year increase in age at seroconversion.

To represent the uncertainty around the *k* parameter, we have assigned a gamma prior distribution with a mean of 0.18 and a standard deviation of 0.06, with 2.5 and 97.5 percentiles at 0.08 and 0.32 respectively. Following the same logic as explained in the previous section, these 2.5 and 97.5 percentiles are consistent with hazard ratios of 1.22 and 1.98 respectively, while the mean of 0.18 is consistent with a hazard ratio of 1.51. The prior distribution is thus consistent with the range of empirical estimates quoted in the previous paragraph. The mean of 0.18 is also consistent when compared with a regression model fitted to the pooled analysis of data from high-income countries , which estimated that the median survival time increased by 19% for each 10-year reduction in age at seroconversion.

### 1.2.4 Extent of HIV viral evolution (*E*)

There has been much speculation that HIV virulence may be changing over time. However, evidence is conflicting. In a systematic review and meta-analysis, Herbeck *et al* found a trend towards significantly declining CD4 counts in early HIV infection over time, and increasing set point viral load (SPVL), with the latter increasing by an average of 0.013 log_10_ copies/ml per year (95% CI: -0.001 to 0.030). Similar results were found in collaborative analyses of data from Europe . This evidence suggests that HIV has evolved towards a more virulent form, over time. However, these studies included only data from high income countries with mainly subtype B epidemics, and the generalizability of these studies to the South African setting is therefore unclear. In a study of South African miners , HIV mortality rates were found to be higher in the post-1993 period than prior to that, though this was only of borderline significance (aHR 1.17, 95% CI: 0.99-1.38). This appears to be consistent with the trend in high income settings.

However, others have argued that HIV may be evolving towards a less virulent form over time . Payne *et al* found when comparing HIV isolates in South Africa and Botswana (which had an earlier start to its HIV epidemic) that there was a greater degree of HLA-driven HIV adaptation in Botswana, and that the Botswanan isolates had lower virulence; in addition, it was found when comparing South African isolates from the 2002-5 and 2012-13 periods that the latter had higher levels of HLA-driven adaptation. In the Rakai district of Uganda, Blanquart *et al* found that SPVL declined by an average of 0.029 log_10_ copies/ml per year (95% CI: 0.013 to 0.045) over the period from 1995 to 2012. In a collaborative analysis of European seroconverter cohorts, Lodi *et al* found that although there was a trend towards lower *initial* CD4 counts in the post-1995 period, there was also a trend towards a slower *rate of decline* in CD4 counts post-1995. The latter is consistent with a trend towards slower HIV disease progression over time.

Given the lack of consistent evidence, we assign a gamma prior distribution to represent the uncertainty regarding the *E* parameter. The mean has been set to 1 (meaning that *a priori*, we consider it equally likely that HIV is becoming more virulent compared to less virulent) and the standard deviation has been set to 0.0065. With these parameters, the 2.5 and 97.5 percentiles of the prior distribution are 0.9873 and 1.0128 respectively. The lower bound corresponds to the lower limit of the Blanquart *et al* study (0.045 per year), assuming the rate of HIV disease progression increases by a factor of 2 for each 1-unit increase in the log_10_ viral load and that the time to disease progression is Weibull-distributed with a shape parameter of 2.5 (i.e. 0.9873^2.5^ = 2^-0.045^). The upper bound exceeds the upper confidence interval limit in the Herbeck *et al* meta-analysis , i.e. 1.0128^2.5^ > 2^0.03^. The reference year in equation (1) is 1999, since 1999 is the median year of death in the study of South African gold miners on which many of our natural history parameters are based .

A limitation of this approach to modelling HIV evolution is that it assumes a constant trend in virulence. In reality, the change in virulence might be more rapid in the early stages of the epidemic, and the trend might not be monotonic .

## 1.3 Mortality in adults on ART

Our model of survival after ART initiation is based on a model that has been fitted to mortality data from six different ART programmes operating in South Africa and participating in the IeDEA Southern Africa collaboration . This relative survival model stratifies mortality rates by age, sex, baseline CD4 category and time since ART initiation. The model also allows for heterogeneity in mortality rates between the six treatment programmes, and estimates HIV-specific mortality by subtracting non-HIV mortality rates estimated in the ASSA2008 model from the crude mortality rates. For those patients who were lost to follow-up, information from the national population register was incorporated to obtain ‘corrected’ estimates of mortality. This relative survival model estimated significant differences in HIV-specific mortality by age and sex, as well as by baseline CD4 count and duration of ART. Table 1 shows the assumed annual mortality rates derived from this model.

Table 1: HIV mortality rates by time since ART initiation, baseline CD4 count and sex

| Time since ART initiation | Baseline CD4 200-349 | | Baseline CD4 <200^*^ | |
| --- | --- | --- | --- | --- |
|  | Men | Women | Men | Women |
| First 6 months of ART (*d* = 0) | 0.0146 | 0.0159 | 0.2168 | 0.1699 |
| Months 6-18 after ART start (*d* = 1) | 0.0132 | 0.0101 | 0.0533 | 0.0416 |
| Months 18-30 after ART start (*d* = 2) | 0.0116 | 0.0057 | 0.0281 | 0.0216 |
| Months 30-42 after ART start (*d* = 3) | 0.0076 | 0.0034 | 0.0185 | 0.0130 |
| >42 months after ART start (*d* = 4) | 0.0063 | 0.0025 | 0.0152 | 0.0094 |

Rates apply to adults aged 30. * Baseline rates that apply when ART initiation rates are close to zero; the mortality rates are scaled down to a theoretical minimum as the rate of ART initiation increases (see Appendix A).

Suppose that *T_g_*,*_d_*,*_c_*(*x*,*t*) represents the annual HIV mortality rate in patients who are in ART duration category *d* in year *t*, of sex *g* and age *x*, who started ART in CD4 category *c*. This is calculated as

$T_{g,d,c}(x,t)=\left\{\begin{matrix} D_{g,d,c}\psi_{g}^{(x-30)/10}I_{d} \text{for} c\geq200 \\ D_{g,d,c}\psi_{g}^{(x-30)/10}I_{d}B(t) \text{for} c<200,d=0 \\ D_{g,d,c}\psi_{g}^{(x-30)/10}I_{d}C(t) \text{for} c<200,d>0 \end{matrix}\right.$ (6)

where the *D_g_*,*_d_*,*_c_* terms are the base rates specified in Table 1, *ψ_g_* is an age adjustment factor, *I_d_* is an adjustment factor to represent possible bias in the IeDEA data, and *B*(*t*) and *C*(*t*) are factors that allow for changes in mortality over time as the fraction of patients starting ART with advanced immune suppression declines.

### 1.3.1 Baseline mortality rates (*D_g_*,*_d_*,*_c_*)

The *D_g_*,*_d_*,*_c_* terms are calculated as the average of the mortality rates estimated by the relative survival model for each of the six cohorts. The relative survival model estimates mortality over four treatment durations (0-12, 12-24, 24-36 and >36 months), which differ from the treatment durations used in the demographic model (0-6, 6-18, 18-30, 30-42 and >42 months). We have set the assumed mortality rate in the first 6 months to be 1.5 times that estimated in months 0-12 in the relative survival model, and we have set the assumed mortality rate in months 6-18 to be the average of half the mortality rate in months 0-12 and the mortality rate in months 12-24. The implicit assumption here is that the mortality rate in the first 6 months on ART is approximately three times the mortality rate in the second 6 months of ART . The assumed mortality rates in the 18-30 and 30-42 month periods are similarly set at the average of the mortality rates estimated over the corresponding durations in the relative survival model.

In the data set to which the relative survival model was fitted, relatively few patients started ART with CD4 counts above 200, because the South African treatment guidelines in place at the time only recommended ART initiation at CD4 counts above 200 if the patient had WHO stage IV symptoms. As symptomatic patients are likely to have higher mortality than asymptomatic patients, even after controlling for CD4 count , the relative survival model estimates of mortality in the baseline CD4 200+ category are likely to exaggerate the future mortality rates in asymptomatic patients starting ART with CD4 counts of 200-349. We have therefore multiplied the relative survival model estimates by factors of 0.2 and 0.5 in the periods up to and after the first 6 months of ART respectively. These multiples are derived from a South African study of ART mortality, which found that after controlling for baseline CD4 count, the ratio of mortality in patients with baseline WHO stage IV to that in patients with no symptoms at baseline dropped from 5.82 in the first 4 months of ART, to 2.78 in months 4-12 and to 1.98 after the first 12 months of ART .

The relative survival model estimates cannot be used to estimate the likely mortality rates in patients starting ART with CD4 counts above 350, as there were too few patients starting ART at these high CD4 counts. Observational data from high income countries suggest that untreated patients with CD4 counts above 250 cells/μl have similar long-term mortality rates, as long as they start ART before their CD4 count declines below 250 cells/μl . We have therefore set the HIV mortality rates of patients starting ART at higher CD4 counts in such a way that the predicted long-term mortality rate in untreated patients with CD4 counts above 500 cells/μl is roughly the same regardless of whether they start ART immediately, defer ART to when their CD4 count drops below 500, or defer ART to when their CD4 count drops below 350. The assumed mortality rates are shown in Table 2.

Table 2: HIV mortality rates in patients starting ART at CD4 counts of 350 cells/μl or higher

| Time since ART initiation | Baseline CD4 ≥500 | | Baseline CD4 350-499 | |
| --- | --- | --- | --- | --- |
|  | Men | Women | Men | Women |
| First 6 months of ART (*d* = 0) | 0.0002 | 0.0001 | 0.0016 | 0.0016 |
| Months 6-18 after ART start (*d* = 1) | 0.0009 | 0.0008 | 0.0050 | 0.0045 |
| Months 18-30 after ART start (*d* = 2) | 0.0027 | 0.0020 | 0.0085 | 0.0057 |
| Months 30-42 after ART start (*d* = 3) | 0.0042 | 0.0027 | 0.0076 | 0.0034 |
| >42 months after ART start (*d* = 4) | 0.0049 | 0.0025 | 0.0063 | 0.0025 |

Rates apply to adults aged 30.

### 1.3.2 Age adjustment factor (*ψ_g_*)

The relative survival model estimated that the increase in male AIDS mortality, per 10-year increase in age, is 1.08 (95% CI: 1.02-1.14) during the first year of ART and 1.13 (95% CI: 1.01-1.28) at longer ART durations. We have set the *ψ*_1_ parameter (for men) to 1.12, which is a weighted average of these two relative hazards (a weight of 0.2 was given to the first-year estimate, based on the finding that the probability of death in the first year after ART initiation was roughly 20% of the lifetime risk of dying from AIDS after ART initiation ). Similarly, the *ψ*_2_ parameter (for women) is set to 1.09, a weighted average of the relative survival model estimates for the first year of ART (1.07, 95% CI: 1.02-1.12) and longer durations (1.09, 95% CI: 0.98-1.22).

### 1.3.3 IeDEA bias adjustment factor (*I_d_*)

The actual mortality rates in South African ART patients may differ from those estimated from the IeDEA-SA data for a number of reasons. We speculate that the bias in the IeDEA-SA estimates may be different at early ART durations and at longer ART durations. This section therefore starts with a discussion of the bias at early ART durations and then proceeds to a discussion of reasons why the bias may differ at longer ART durations.

*IeDEA-SA bias during the first 6 months after ART initiation*

Any IeDEA-SA bias that exists in the first 6 months on ART is likely to be due mainly to better functioning of services in IeDEA clinics compared to the average level of services in South African clinics. In a best case scenario, where there is no heterogeneity in mortality rates across ART programmes within South Africa, we would expect the ratio of average mortality across South African ART services to that in the IeDEA-SA cohorts to be 1. In a worst case scenario, we might expect the ratio to be approximated by the ratio of the average mortality rate in a representative sample of South African ART services to the lowest mortality rate in that sample. To represent the uncertainty around the true ratio, we aim to set a gamma prior distribution, with 2.5 and 97.5 percentiles corresponding to the ratios in the best and worst case scenarios respectively.

Estimating the ratio in the worst case scenario presents a challenge. Unfortunately there are no representative published estimates of the extent of variation in mortality rates across South African ART programmes. However, there have been a few studies that have reported the extent of variation in mortality rates or attrition rates (a crude proxy for mortality) across different clinics within selected groups of South African ART programmes (Table 3). In these three studies, the ratio of average mortality to minimum mortality varied between 1.9 and 8.1. In two of the studies, the measures being compared did not control for differences between programmes in baseline patient characteristics , which may have led to the extent of the variation across programmes being overstated. However, one study considered only IeDEA-SA cohorts and one study considered only private and NGO cohorts , which may have led to the extent of the variation being understated. It is also worth noting that even if all ART programmes were identical, stochastic variation would cause the observed ratios to be above one, i.e. leading to over-estimation of the extent of variation. Given these various biases, we consider an upper bound of 3 on the ratio to be most plausible. We have therefore set the mean and standard deviation of the gamma prior distribution on the *I*_0_ parameter to 1.85 and 0.5 respectively, which yields 2.5 and 97.5 percentiles at 1.0 and 3.0 respectively.

Table 3: South African studies comparing mortality rates across ART cohorts

| Study | Cohort description | Ratio of average  mortality to  minimum mortality |
| --- | --- | --- |
| Charalambous *et al* | 36 private and NGO clinics participating  in Aurum Institute HIV programme | 3.05 |
| Vella *et al* | 32 public clinics in KwaZulu-Natal | 8.1^*^ |
| Johnson *et al* | 6 cohorts participating in IeDEA-SA  collaboration |  |
|  | Males, 1^st^ 6 months on ART | 1.89 |
|  | Females, 1^st^ 6 months on ART | 1.85 |

* Combined mortality and loss to follow-up.

*IeDEA-SA bias at durations longer than 42 months after ART initiation*

The bias that exists in the open interval (more than 42 months after ART initiation) might be expected to be different from that in the 0-6 month interval, for two reasons. The first reason is that many patients move out of the IeDEA-SA site in which they started ART, either as a result of ART discontinuation or transfer to another ART service. Any advantage to receiving ART in an IeDEA-SA site would therefore diminish over time (taking into account that the vital registration system continues to record deaths after patients have left the original IeDEA-SA clinic). Cornell *et al* estimated a retention rate of 60% in IeDEA-SA cohorts by 48 months after ART initiation and 56% by 60 months. If *I*_0_ is the IeDEA-SA bias in the first 6 months after ART initiation, if rates of dropout from IeDEA-SA programmes are the same as those in the national ART programme, and if individuals who have dropped out of IeDEA-SA cohorts have the same mortality rates on average as other South African ART patients, then the IeDEA-SA bias that might be expected at 48 months is approximately 1 + 0.6 × *I*_0_. This may be an under-estimate of the bias, because not all of the remaining 40% are still alive. It might also be an under-estimate because we have shown previously that the retrospective definition of loss to follow-up that was used by Cornell *et al* is biased towards over-stating the extent of loss to follow-up . This is because many of the individuals who are classified lost to follow-up in the short term ultimately return to care at IeDEA-SA sites.

The second reason for a different bias at longer durations is that the relative survival model used to estimate HIV-specific mortality was fitted on the assumption of piecewise-constant hazards over different ART durations. This will tend to exaggerate mortality rates if follow-up times are short, because if mortality rates are continuously decreasing with respect to ART duration, and follow-up is concentrated at the lower end of the open interval, the estimated average mortality rate over the open interval will be biased towards the higher mortality at the lower end of the interval . To illustrate this, we refitted the relative survival model using cubic splines to represent the baseline hazard, using the *stpm2* command in STATA 13 . The resulting estimates of life expectancies were compared to those obtained when using the piecewise-constant modelling approach applied to the same dataset . Fig B compares the life expectancies of patients starting ART in the 2007-9 period (similar results were obtained when considering other ART initiation periods). The results show that the use of the cubic spline model generally yields higher estimates of life expectancy than the piecewise-constant model, especially in individuals starting ART at young ages and at low CD4 counts. This confirms that the mortality rates estimated from the application of the piecewise-constant model could be over-estimates.

Fig B: Life expectancies of patients starting ART, by age at ART initiation

Error bars represent 95% confidence intervals.

To get a lower bound on the bias that exists due to the piecewise-constant assumption, we can consider the ratio of the HIV-specific mortality 8 years after ART initiation to that 3.5 years (42 months) after ART initiation, as estimated by the cubic spline model. This is a lower bound because (a) we are modelling HIV mortality in the period up to the end of 2014, and over this period, the average time since ART initiation, for those ART patients in the >42 months duration interval, is likely to be less than 8 years; and (b) we are estimating mortality in the >42 month interval from IeDEA-SA data in the >36 month interval, and the average duration of follow-up in the >36 month interval is likely to be more than 6 months. Assuming a difference of 5.5 years between the modelled average ART duration and the actual average ART duration in the open interval is therefore very conservative. Table 4 summarizes the ratio of the HIV-specific mortality 8 years after ART initiation to that 3.5 years after ART initiation, as estimated by the cubic spline model. Based on the values in Table 4, we assume a lower bound on the bias due to piecewise-constant assumption of 0.65.

Table 4: Cubic spline estimates of HIV-specific mortality rate ratio comparing 8 years after ART initiation to 3.5 years after ART initiation

|  | Baseline CD4 <50 | | Baseline CD4 200+ | |
| --- | --- | --- | --- | --- |
|  | Males | Females | Males | Females |
| Mortality ratio | 0.625 | 0.664 | 0.653 | 0.719 |

Now consider the ratio *J* = *I*_4_/*I*_0_. If the two sources of bias described previously are negligible, we would expect this ratio to be 1; this is assumed to be an upper bound on *J*. However, if we consider the lower bound due to each source of bias, a lower bound on *J* is

$$\frac{0.65\times\left(1+0.6I_{0}\right)}{I_{0}}$$

$=0.65\times\left(\frac{1}{I_{0}}+0.6\right)$. (7)

Since we are trying to find a lower bound on this ratio, and since we have previously assumed that a plausible upper bound on *I*_0_ is 3, substitution of *I*_0_ = 3 into this equation yields a lower bound of 0.61. Thus we specify a gamma prior distribution to represent the uncertainty regarding the ratio *J*, with a mean of 0.8 and a standard deviation of 0.1; this prior distribution has its 2.5 and 97.5 percentiles at 0.62 and 1.01 respectively, roughly corresponding to the lower and upper bounds that we consider plausible.

### 1.3.4 Time-dependent adjustment factors (*B*(*t*) and *C*(*t*))

Although the relative survival model calculates rates of mortality for four baseline CD4 categories (<50, 50-99, 100-199 and 200+), our demographic model uses these to estimate mortality rates in only two CD4 categories (<200 and 200-349). The baseline assumptions shown in Table 1 for the CD4 <200 category are calculated as a weighted average of the mortality estimates in each of the <50, 50-99 and 100-199 categories, with the weights calculated on the assumption of a baseline CD4 distribution that is uniform on the interval [0, 200). Although this uniform assumption is reasonable in the very early stages of the ART rollout , we would expect a change in the distribution of baseline CD4 counts among individuals starting ART at CD4 <200, as rates of ART initiation increase and as there are fewer untreated individuals remaining at CD4 counts below 50 . We have therefore created a sub-model to evaluate the likely change in the disease severity of patients with CD4 <200 over time, as a function of the average rate of ART initiation. This sub-model is used to derive adjustments both to the rate of untreated mortality at CD4 <200 and to the rate of mortality in patients who started ART with CD4 <200. A detailed description of this sub-model is provided in Appendix A. Briefly, we specify a parameter *m*, which determines the reduction in excess mortality (on a log scale, relative to a theoretical minimum) per unit increase in the average rate of ART initiation at CD4 counts <200 cells/μl, over the previous three years. Mathematically,

$B(t)=0.39+\left(1-0.39\right)\exp\left(-mr_{t-}\right)$, (8)

where 0.39 is the ratio of the theoretical minimum mortality to that at the start of ART rollout (Table 1), and $r_{t-}$ is the average rate of ART initiation over the three years prior to year *t*. Similarly,

$C(t)=0.61+\left(1-0.61\right)\exp\left(-mr_{t-}\right)$. (9)

A higher ratio (0.61) is estimated here because the baseline CD4 count is less predictive of mortality after the first 6 months of ART than during the first 6 months of ART, and hence *C*(*t*) is less sensitive to changes over time in the baseline CD4 distribution. A prior distribution is specified to represent the uncertainty regarding the *m* parameter. Further explanation is provided in Appendix A.

## 1.4 Modelling of HIV diagnosis and ART initiation

Individuals are assumed to be diagnosed at a monthly rate that depends on their age, sex, HIV stage and HIV testing history, and these monthly rates change over time. HIV-positive adults are assumed to be diagnosed through one of three HIV testing modalities: antenatal HIV testing, HIV testing of patients with opportunistic infections (OIs) and other HIV testing modalities. Antenatal HIV testing rates are set based on women’s assumed age-specific rates of fertility (adjusted to take into account the effect of HIV on fertility) and public sector statistics on the proportions of pregnant women receiving HIV testing . The rate of testing as a result of OIs is assumed to depend on the incidence of OIs by HIV stage and public sector estimates of the fraction of OI patients tested for HIV . Finally, the incidence of testing through other modalities is determined by calibrating the model to total recorded numbers of HIV tests performed in South Africa (public and private sectors combined), with household survey data on history of HIV testing being used to determine relative rates of test uptake by age and sex . The model also assumes that individuals who have previously tested for HIV have a higher rate of testing than individuals who have never previously tested, though HIV-diagnosed individuals are assumed to be less likely to get retested. A more detailed description of the model of HIV diagnosis has been published previously .

The modelling of adult ART initiation has been described previously . Briefly, adult ART initiation is modelled as occurring either (1) at the time of HIV diagnosis (if the individual is eligible to start ART), or (2) at a later stage, after diagnosis.

In the case of adults who are newly diagnosed, the probability of ART initiation is assumed to depend on the setting in which the patient is diagnosed: uptake is assumed to be higher in pregnant women and patients who are screened for HIV when symptomatic than in patients who are non-pregnant and asymptomatic . The probability of ART initiation following diagnosis is assumed to also depend on the guidelines in place at the time of diagnosis. Table 5 shows the proportions of patients who are assumed to be eligible, in different categories. In some of the periods the assumed eligible proportion has been set to 50% because the change in guideline occurred midway through the relevant period. For patients with CD4 counts of 200-349 cells/μl, the model allows for non-zero access to ART prior to official guideline changes, as some NGO-supported programmes and private sector programmes applied higher CD4 eligibility thresholds . The proportions are further adjusted to allow for the possibility that – even when official guidelines indicate that an individual is eligible – the individual might not start ART because of ART not being available at the patient’s nearest clinic.

Table 5: Proportions of adult patients assumed to be eligible to receive lifelong ART

|  | 2000-  2003^*^ | 2003-  2009 | 2009-  2010 | 2010-  2011 | 2011-  2012 | 2012-  2014 | 2014-  2015 |
| --- | --- | --- | --- | --- | --- | --- | --- |
| WHO stage IV or CD4 <200 | 100% | 100% | 100% | 100% | 100% | 100% | 100% |
| Pulmonary TB, CD4 200-349 | 10% | 10% | 50% | 100% | 100% | 100% | 100% |
| WHO stage III, CD4 350+ | 0% | 0% | 0% | 0% | 0% | 100% | 100% |
| Pregnant women, CD4 200-349 | 10% | 10% | 50% | 100% | 100% | 100% | 100% |
| Pregnant women, CD4 350+ | 0% | 0% | 0% | 0% | 0% | 0% | 50% |
| Asymptomatic, non-pregnant, CD4 200-349 | 10% | 10% | 10% | 20% | 80% | 100% | 100% |
| Asymptomatic, non-pregnant, CD4 350-499 | 0% | 0% | 0% | 0% | 0% | 0% | 50% |
| Asymptomatic, non-pregnant, CD4 500+ | 0% | 0% | 0% | 0% | 0% | 0% | 0% |

Calendar periods are defined to run from the middle of the first year quoted to the middle of the second year.

* Applies only to rollout in private sector and NGO-run programmes.

In each year, the estimated number of adult men starting ART at the time of diagnosis is subtracted from the estimated total number of men starting ART in order to obtain the number of men starting ART after diagnosis. The rate at which ART is initiated in previously-diagnosed men is then calculated based on the number of eligible diagnosed and untreated men, allowing for differences by CD4 category . (The same calculation is performed for women.)

Fig C shows that this approach leads to model estimates of total numbers on ART consistent with reports from the public sector (the model estimates have been adjusted downward to exclude private sector provision, based on biennial surveys of ART provision in the private and NGO sectors ). Prior to 2009, almost all public sector reports on the South African ART programme reported *cumulative* ART enrolment, but from September 2009, most provinces gradually switched to reporting *current* ART enrolment; for this reason, Fig C includes model estimates of both cumulative enrolment (consistent with reported data up to September 2009) and current enrolment (consistent with reported data from 2012 onward).

Fig C: Model calibration to ART programme statistics

Model estimates are shown for both current ART enrolment and cumulative ART enrolment, because reporting of public sector totals gradually changed from cumulative to current after September 2009. Model estimates have been adjusted to exclude private sector estimates.

# 2. Model of HIV transmission

The model of HIV transmission has been described in detail elsewhere . Briefly, sexually active adults are divided into two broad groups: high risk (defined as individuals with a propensity for concurrent partnerships and commercial sex) and low risk (individuals who are monogamous). Within each class, individuals are divided into further sub-groups that are defined in terms of marital status and (if married) their partner’s risk group. Men are classified according to whether or not they are circumcised. Three types of relationship are modelled: cohabiting/marital relationships, short-term relationships, and interactions between sex workers and their clients. Rates of marriage are assumed to depend on the individual’s age and sex, while rates of short-term partnership formation depend on age, sex, risk group and marital status (low risk individuals, by definition, do not engage in short-term relationships while married). Coital frequencies are assumed to depend on age, relationship type, and stage of HIV infection (assuming that individuals in the later stages of HIV infection are less likely to engage in sexual activity). Rates of condom use are assumed to depend on age, sex and relationship type, and change over time in response to HIV awareness campaigns. In addition, HIV-positive individuals are assumed to increase their level of condom usage after diagnosis and after ART initiation.

The probability of HIV transmission per act of sex depends on the HIV stage of the HIV-positive partner and whether they are on ART, the type of the relationship, and the age and sex of the susceptible partner. The parameter $\beta_{g,l}$ is defined as the average HIV transmission probability, in a single act of unprotected sex, from an untreated HIV-positive individual of sex *g*, to an HIV-negative partner in relationship type *l* (before adjusting for differences in transmission probabilities by HIV stage and age). Transmission probabilities are assumed to be reduced when condoms are used, and men are assumed to have a lower risk of HIV acquisition if they are circumcised. A specified initial HIV prevalence in high risk women in 1985 (*V*_0_) acts as the parameter that ‘seeds’ the HIV epidemic in South Africa.

The model of HIV transmission in the present analysis is the same as that described previously , but with one modification: it is assumed that if there is a change in HIV virulence over time (i.e. *E* ≠ 1), there would be associated changes in HIV transmission probabilities. Changes in virulence and infectivity would both be driven by changes in set point viral load (SPVL) , so it is reasonable to assume that such an association exists, although some experiments suggest that less virulent HIV strains are not necessarily less transmissible . If *Y* is the annual change in HIV transmission probability due to changes in HIV virulence, then the average transmission probability that applies in year *t* is

$\beta_{g,l}Y^{t-1999}$, (10)

assuming that the base transmission probabilities apply in 1999 (to be consistent with equation (1)). Now suppose that *θ* is the average annual change in SPVL (on a log_10_ scale) due to viral evolution, and that this is responsible for the change in virulence as well as the change in infectiousness. Further suppose that *F* is the factor by which the probability of HIV transmission increases per unit increase in log viral load. Then

$Y=F^{\theta}$. (11)

Similarly, we define *P* as the factor by which the rate of HIV disease progression increases per unit increase in log viral load (HIV disease progression can be thought of as the rate of progression to AIDS or CD4 <200/μl in the absence of ART). We define *α* as the ratio $\ln(F)/\ln(P)$, i.e. the ratio of the increase in infectivity to the increase in HIV disease progression (on a natural log scale), for a given change in SPVL. If the time to HIV disease progression is approximately Weibull-distributed with a shape parameter of *ϕ*, then

$E^{\varphi}=P^{\theta}$, (12)

because a Weibull hazard is inversely proportional to the mean, raised to the power of the shape parameter, and hence a reduction in the mean by a factor of *E* implies an increase in the hazard by a factor of $E^{\varphi}$. From the previous equation it follows that

$\theta=\varphi\frac{\ln(E)}{\ln(P)}$. (13)

Substituting this into equation (11) gives

$$Y=F^{\varphi\ln(E)/\ln(P)}$$

$$=\exp\left(\ln(F)\varphi\ln(E)/\ln(P)\right)$$

$=E^{\alpha\varphi}$. (14)

Since we have already specified a prior distribution on the *E* parameter, it remains to determine plausible values for the *α* and *ϕ* parameters. The shape parameter, *ϕ*, has been fixed at 2.5, based on studies that involved fitting Weibull distributions to HIV survival times in the pre-ART era . The *α* parameter is more difficult to determine precisely. The increase in HIV transmission risk per unit increase in log SPVL (*F*) is estimated to be between 1.8 and 2.9 (average of 2.4) , while the increase in HIV disease progression per unit increase in log SPVL (*P*) is estimated to be between 1.8 and 3.2 (also an average of 2.4) . It therefore seems reasonable to model the uncertainty in *α* by assigning a gamma prior with a mean of 1. A likely lower limit on the ratio is 0.5 (ln(1.8)/ln(3.2)), while a likely upper limit is 1.8 (ln(2.9)/ln(1.8)). We have therefore chosen a standard deviation of 0.4 for the prior distribution, which gives 2.5 and 97.5 percentiles at 0.38 and 1.92 respectively.

All but four of the HIV transmission parameters have been fixed at the posterior means estimated previously , in order to reduce the number of free parameters in the model fitting procedure and thus ensure convergence of the model fitting procedure. The four exceptions that have been made are the *α* parameter described previously, the initial HIV prevalence in high risk women (*V*_0_), the average male-to-female transmission probability in short-term relationships ($\beta_{1,0}$) and the average female-to-male transmission probability in short-term relationships ($\beta_{2,0}$). These parameters were allowed to vary in order to ensure that the model maintained an acceptable fit to the HIV prevalence data as the HIV survival parameters were adjusted. Very roughly, the *V*_0_ parameter determines the timing of when the epidemic starts, the $\beta_{1,0}$ and $\beta_{2,0}$ parameters determine how rapidly HIV prevalence increases in the early phase of the epidemic (and the relative levels of HIV prevalence in men and women), and the *α* parameter determines how rapidly HIV incidence declines in the more advanced stages of the HIV epidemic. The prior distributions assigned to the *V*_0_, $\beta_{1,0}$ and $\beta_{2,0}$ parameters are the same as described previously .

# 3. Model fitting procedure

We adopt a Bayesian approach to model fitting and uncertainty analysis. This involves specifying prior distributions to represent the uncertainty around the key HIV parameters, specifying a likelihood function to represent how well the model fits the vital registration and HIV prevalence data (for a given set of input parameters), and estimating the posterior distribution of parameters that give the best fit to the data, given the constraints on the input parameters. The following sections describe each of these steps in more detail.

## 3.1 Prior distributions

The prior distributions and the data sources on which they are based have been described in previous sections. Table 6 summarizes the prior distributions from previous sections. In addition, we have added a prior for the reduction in unprotected sex that occurs after HIV diagnosis; this parameter is only included in alternative analyses 1 and 2 (described in the main text). The reason for including this additional parameter is that in alternative analyses 1 and 2, the model has less flexibility to match the observed change in HIV prevalence over time (because we are no longer allowing for the changes in HIV transmissibility associated with the changes in HIV virulence). To compensate for this, we allow the effect of HIV diagnosis on condom use to vary in the model fitting, since this parameter is influential in determining the model trend in HIV prevalence over time. The prior distribution on this parameter is the same as that assumed previously .

Table 6: Prior distributions

| Parameter | Symbol | Prior | Mean | Standard  deviation |
| --- | --- | --- | --- | --- |
| Average survival in absence of ART (years) | *π* | Gamma (144, 12) | 12 | 1 |
| RR of HIV disease progression in women | *ϖ* | Gamma (369, 384) | 0.96 | 0.05 |
| Increase in HIV disease progression per  10-year increase in age | *k* | Gamma (9, 50) | 0.18 | 0.06 |
| RR of HIV disease progression per calendar year | *E* | Gamma (23669, 23669) | 1 | 0.0065 |
| IeDEA-SA bias <6 months after ART start | *I*_0_ | Gamma (13.69, 7.40) | 1.85 | 0.50 |
| Ratio of IeDEA-SA bias >42 months after ART  start to bias <6 months after ART start | *J* | Gamma (64.0, 80.0) | 0.80 | 0.10 |
| Reduction in mortality* per unit increase in rate of  ART initiation (at CD4<200) over last 3 years | *m* | Gamma (4.59, 0.612) | 7.5 | 3.5 |
| Ratio of increase in infectivity* to increase in  disease progression,* per unit change in SPVL | *α* | Gamma (6.25, 6.25) | 1 | 0.4 |
| Initial HIV prevalence in high risk women | *V*_0_ | Uniform (0, 0.002) | 0.001 | 0.00058 |
| Male-to-female transmission probability in short-  term relationships | *β*_1,0_ | Beta (5.68, 468) | 0.012 | 0.005 |
| Female-to-male transmission probability in short-  term relationships | *β*_2,0_ | Beta (7.05, 874) | 0.008 | 0.003 |
| Reduction in unprotected sex after HIV diagnosis† | - | Beta (5.90, 2.77) | 0.68 | 0.15 |

* On a natural log scale. † Only considered in alternative analyses 1 and 2, not in the main analysis. IeDEA-SA = International epidemiology Databases for the Evaluation of AIDS, Southern Africa. RR = relative risk. SPVL = set point viral load.

## 3.2 Likelihood function

The model is calibrated to three data sources: recorded death data, antenatal clinic survey data and household survey data. The likelihood for all three data sources is the product of the likelihood calculated for each individual data source, as detailed below. All parameters are estimated jointly with respect to all of the available data, although the likelihood is presented separately for each data source in the sections that follow.

### 3.2.1 Recorded death data

To calculate the likelihood in respect of the reported death data, we restrict this analysis to deaths occurring over the period from the start of 1997 to the end of 2014 . Because cause of death information is seldom captured accurately, and reported AIDS deaths are likely to be only a fraction of the actual HIV-related deaths , we compare model estimates of all-cause mortality with reported levels of all-cause mortality. This comparison is only likely to be meaningful in those age groups in which a substantial proportion of deaths are HIV-related, and this analysis is therefore restricted to deaths occurring from ages 20 to 59. Mortality data are grouped in 5-year age bands for calibration purposes, and estimates are considered separately for males and females.

Suppose that $\Theta_{g,x,t}(\mathbf{\varphi})$ represents the model estimate of the number of deaths in individuals of sex *g*, between ages *x* and *x* + 4, in year *t*, where the vector $\mathbf{\varphi}$ represents the values of the model input parameters. Further suppose that $R_{g,x,t}$ represents the reported number of deaths in individuals of sex *g*, between ages *x* and *x* + 4, in year *t*. In order to specify a likelihood function for the reported death data, it must be assumed that a certain proportion of adult deaths, *γ_g_*,*_x_*,*_t_*, is reported. It is assumed that if $\mathbf{\varphi}$ is the true set of parameter values, then the difference between the log-transformed model estimate of the number of reported deaths ($\Theta_{g,x,t}(\mathbf{\varphi})\gamma_{g,x,t}$) and the log-transformed actual number of reported deaths is normally distributed with zero mean. More formally, the likelihood is calculated on the assumption that

$\log\left(R_{g,x,t}\right)=\log\left(\Theta_{g,x,t}(\mathbf{\varphi})\gamma_{g,x,t}\right)+\varepsilon_{g,x,t}$, (15)

where $\varepsilon_{g,x,t}\sim N\left(0,\sigma_{d}^{2}\right)$. The parameter $\varepsilon_{g,x,t}$ can be regarded as comprising both a ‘model error’ and ‘random binomial error’ component, but because the population numbers are very large, the random binomial component of the error is relatively small on the log scale. It is therefore reasonable to assume that the variance of the error term is independent of the population size in the relevant sex and age group.

The *γ_g_*,*_x_*,*_t_* parameters have been estimated from a variety of sources. Over the period from October 1996 to October 2001, Dorrington *et al* estimate that the fraction of adult deaths recorded was 84%, based on death distribution methods (i.e. based on comparing the recorded numbers of adult deaths to the changes in the population sizes in each age cohort over the inter-census period). The authors also estimate that the annual increase in the proportion of deaths recorded, over this 5-year period, was 1.7% in men and 2.1% in women, based on an assumption of stable mortality rates at ages 65 and older (where AIDS would be expected to have relatively little impact on mortality). In the period after 2001, estimates of the completeness of adult death recording have been around 93%, based on similar methods . Based on these estimates, we set initial completeness assumptions – independent of age and sex – that increase linearly from 80.2% in 1997 to 87.8% in 2001 (an increase of 1.9% per annum, with 84% completeness in 1999) and 93% in 2004, after which completeness is assumed to remain constant (Table 7). The assumption of constant completeness after 2004 is supported by an analysis of factors affecting the recording of deaths in ART patients, which showed no significant change in the completeness of vital registration over the 2004-2014 period .

In the final set of completeness assumptions, we use the completeness estimates by age and sex, as estimated in the analysis of factors affecting the recording of deaths in ART patients over the 2004-2014 period , and scale these down by the ratio of initial completeness assumptions to 0.93 in the period prior to 2004. The completeness assumptions are shown in Table 7. Although these ‘final’ completeness assumptions have the advantage of taking into account known age and sex differences in the recording of mortality, these estimates may be biased because they are based on a select group of patients receiving ART in public-sector clinics, who had ID numbers, and who might not be typical of the general population. We therefore include an alternative analysis in which the completeness adjustments are based only on the initial completeness assumptions, with no adjustment for age and sex effects (Model 3 described in section 3.4). The completeness assumptions in this alternative analysis are close to those in the Second National Burden of Disease Study .

Table 7: Completeness assumptions (fraction of deaths that are recorded)

| Year | 1997 | 1998 | 1999 | 2000 | 2001 | 2002 | 2003 | 2004+ |
| --- | --- | --- | --- | --- | --- | --- | --- | --- |
| Initial completeness assumptions | | | | | | | | |
|  | 0.802 | 0.821 | 0.84 | 0.859 | 0.878 | 0.897 | 0.914 | 0.930 |
| Final completeness assumptions | | | | | | | | |
| Women aged |  |  |  |  |  |  |  |  |
| 20-24 | 0.798 | 0.817 | 0.836 | 0.855 | 0.874 | 0.892 | 0.909 | 0.925 |
| 25-29 | 0.809 | 0.828 | 0.847 | 0.866 | 0.886 | 0.905 | 0.922 | 0.938 |
| 30-34 | 0.817 | 0.836 | 0.855 | 0.875 | 0.894 | 0.913 | 0.931 | 0.947 |
| 35-39 | 0.823 | 0.842 | 0.862 | 0.881 | 0.901 | 0.920 | 0.937 | 0.954 |
| 40-44 | 0.827 | 0.847 | 0.866 | 0.886 | 0.905 | 0.925 | 0.943 | 0.959 |
| 45-49 | 0.831 | 0.850 | 0.870 | 0.890 | 0.909 | 0.929 | 0.947 | 0.963 |
| 50-54 | 0.834 | 0.853 | 0.873 | 0.893 | 0.913 | 0.932 | 0.950 | 0.967 |
| 55-59 | 0.836 | 0.856 | 0.876 | 0.895 | 0.915 | 0.935 | 0.953 | 0.969 |
| Men aged |  |  |  |  |  |  |  |  |
| 20-24 | 0.756 | 0.774 | 0.792 | 0.810 | 0.828 | 0.846 | 0.862 | 0.877 |
| 25-29 | 0.772 | 0.791 | 0.809 | 0.827 | 0.845 | 0.864 | 0.880 | 0.896 |
| 30-34 | 0.789 | 0.807 | 0.826 | 0.845 | 0.863 | 0.882 | 0.899 | 0.914 |
| 35-39 | 0.802 | 0.821 | 0.840 | 0.859 | 0.878 | 0.897 | 0.914 | 0.930 |
| 40-44 | 0.813 | 0.832 | 0.852 | 0.871 | 0.890 | 0.909 | 0.927 | 0.943 |
| 45-49 | 0.821 | 0.841 | 0.860 | 0.880 | 0.899 | 0.918 | 0.936 | 0.952 |
| 50-54 | 0.827 | 0.847 | 0.866 | 0.886 | 0.906 | 0.925 | 0.943 | 0.959 |
| 55-59 | 0.832 | 0.851 | 0.871 | 0.891 | 0.910 | 0.930 | 0.948 | 0.964 |

The maximum likelihood estimate of the parameter $\sigma_{d}^{2}$ is calculated as

$\hat{\sigma}_{d}^{2}=\frac{1}{288}\sum_{g} \sum_{x} \sum_{t=1997}^{2014} \left[\log\left(R_{g,x,t}\right)-\log\left(\Theta_{g,x,t}(\mathbf{\varphi})\gamma_{g,x,t}\right)\right]^{2}$. (16)

The likelihood in respect of the reported death data is then calculated based on the assumed normality of the error terms:

, (17)

where **R** represents the matrix of reported death data.

### 3.2.2 Antenatal HIV prevalence data

The model is fitted to antenatal HIV prevalence data from national surveys that have been conducted from 1997 to 2013 (survey data collected prior to 1997 have not been included, as these early antenatal surveys were based on convenience samples and reported 95% confidence intervals did not include survey design effects). We include HIV prevalence estimates for 5 age groups (15-19, 20-24, 25-29, 30-34 and 35-39).

Suppose that $H_{x,t}\left(\mathbf{\varphi}\right)$ is the model estimate of HIV prevalence in pregnant women aged *x* to *x* + 4, in year *t*, where the vector $\mathbf{\varphi}$ represents the values of the model input parameters. The corresponding prevalence of HIV actually measured in the antenatal survey is represented by $y_{x,t}$. It is assumed that if $\mathbf{\varphi}$ is the true set of parameter values, then the difference between the logit-transformed model estimate and the logit-transformed observed prevalence is normally distributed. The mean of this normal distribution represents the extent of antenatal bias, which arises due to the exclusion of women receiving private antenatal care from the sample and other behavioural factors. The variance of the distribution is assumed to be composed of a ‘survey error’ term (representing the uncertainty around the survey estimate due to binomial variation and cluster variation in the survey) and a ‘model error’ term (representing the error that may arise due to the assumption that the antenatal bias is constant over time and constant with respect to age). More formally, it is assumed that

$\log\left(\frac{y_{x,t}}{1-y_{x,t}}\right)=\log\left(\frac{H_{x,t}\left(\mathbf{\varphi}\right)}{1-H_{x,t}\left(\mathbf{\varphi}\right)}\right)+\eta+u_{x,t}+\varepsilon_{x,t}$, (18)

where *η* is the antenatal bias parameter, $u_{x,t}\sim N\left(0,\sigma_{u}^{2}\right)$ and $\varepsilon_{x,t}\sim N\left(0,\sigma_{x,t}^{2}\right)$. The latter two terms represent the model error and the survey error respectively. The model error term is introduced to account for the possibility that the assumption of a constant antenatal bias may be unrealistic (in reality the antenatal bias may change over time, and might not be the same at all ages). The logit transformations ensure that the error terms are closer to normality and that the model error terms are roughly independent of the level of HIV prevalence. For a given parameter combination $\mathbf{\varphi}$, the antenatal bias parameter is estimated using the formula

$\hat{\eta}=\frac{1}{85}\sum_{x} \sum_{t=1997}^{2013} \left(\log\left(\frac{y_{x,t}}{1-y_{x,t}}\right)-\log\left(\frac{H_{x,t}\left(\mathbf{\varphi}\right)}{1-H_{x,t}\left(\mathbf{\varphi}\right)}\right)\right)$. (19)

The $\sigma_{x,t}^{2}$ values are estimated from the 95% confidence intervals that have been published for the various survey estimates. Once these $\sigma_{x,t}^{2}$ values have been obtained, the $\sigma_{u}^{2}$ parameter is estimated using the formula

. (20)

The likelihood in respect of the antenatal data is then calculated based on the assumption that the error terms are normally distributed:

, (21)

where $\mathbf{y}$ represents the matrix of $y_{x,t}$ values, across age bands 15-19 to 35-39, and across calendar years 1997 to 2013.

### 3.2.3 Household survey HIV prevalence data

The model is calibrated to HIV prevalence data from three nationally-representative household surveys conducted by the Human Sciences Research Council (HSRC) in 2005 , 2008 and 2012 . HIV prevalence levels in each survey are estimated by 5-year age group (from 15-19 up to 55-59) and by sex. The approach adopted in defining the likelihood function in respect of the HSRC HIV prevalence data is the same as that for the antenatal data, except that the bias term (*η*) and model error term (*u*) are both omitted. The omission of the bias term is consistent with the approach adopted in other uncertainty analyses of HIV data in developing countries , in which it is assumed that household prevalence data provide an unbiased estimate of HIV prevalence in the general population. The model error term is omitted because it is not necessary if the survey estimates are truly unbiased (the model error term was introduced in the previous section only because the assumption of a constant antenatal bias might be unrealistic). The total likelihood in respect of the HSRC household survey data is represented by the symbol $L_{3}\left(\mathbf{\Omega}|\mathbf{\varphi}\right)$, where $\mathbf{\Omega}$ represents the matrix of HSRC survey HIV prevalence estimates.

## 3.3 Posterior simulation

The posterior distribution, $p\left(\mathbf{\varphi}|\mathbf{R},\mathbf{y},\mathbf{\Omega}\right)$, represents the synthesis of the prior beliefs regarding the parameters in the vector $\mathbf{\varphi}$ (i.e. the prior distributions summarized in Table 6, which we represent by $p\left(\mathbf{\varphi}\right)$), and the likelihood function. By Bayes’ theorem, the posterior distribution is calculated as

$p\left(\mathbf{\varphi}|\mathbf{R},\mathbf{y},\mathbf{\Omega}\right)=K\times p\left(\mathbf{\varphi}\right)\times L_{1}\left(\mathbf{R}|\mathbf{\varphi}\right)\times L_{2}\left(\mathbf{y}|\mathbf{\varphi}\right)\times L_{3}\left(\mathbf{\Omega}|\mathbf{\varphi}\right)$, (22)

where *K* is a constant, calculated in such a way that the integral of the posterior distribution is 1. Because the calculation of the likelihood function requires us to run a complex mathematical model, there is no closed-form analytical solution to this equation, i.e. there is no simple formula for calculating $p\left(\mathbf{\varphi}|\mathbf{R},\mathbf{y},\mathbf{\Omega}\right)$ for given values of $\mathbf{\varphi}$. It is therefore necessary to use numerical methods to approximate $p\left(\mathbf{\varphi}|\mathbf{R},\mathbf{y},\mathbf{\Omega}\right)$.

The numerical method used in this analysis is Incremental Mixture Importance Sampling (IMIS) . This method was chosen over standard software packages such as BUGS because it was programmed in C++ and could therefore be easily integrated into the existing C++ model. The method we use to generate a posterior sample of parameter combinations follows these steps:

1. Randomly sample 10 000 parameter combinations from the prior distribution $p\left(\mathbf{\varphi}\right)$.
2. For each parameter combination sampled, $\mathbf{\varphi}_{i}$, run the model and calculate the likelihood function $L_{1}\left(\mathbf{R}|\mathbf{\varphi}\right)\times L_{2}\left(\mathbf{y}|\mathbf{\varphi}\right)\times L_{3}\left(\mathbf{\Omega}|\mathbf{\varphi}\right)$.
3. Calculate weights for each of the 10 000 parameter combinations, where the weight for the *i*^th^ parameter combination is $W_{i}=\frac{L_{1}\left(\mathbf{R}|\mathbf{\varphi}_{i}\right)L_{2}\left(\mathbf{y}|\mathbf{\varphi}_{i}\right)L_{3}\left(\mathbf{\Omega}|\mathbf{\varphi}_{i}\right)}{\sum_{j} L_{1}\left(\mathbf{R}|\mathbf{\varphi}_{j}\right)L_{2}\left(\mathbf{y}|\mathbf{\varphi}_{j}\right)L_{3}\left(\mathbf{\Omega}|\mathbf{\varphi}_{j}\right)}$.
4. Form a new multivariate normal sampling distribution, using the parameter combination with maximum weight from the previous step as the mean and calculating the weighted covariance matrix from the 1 000 parameter combinations that are closest to the parameter combination with maximum weight.
5. Randomly sample 1 000 parameter combinations from this new multivariate normal distribution and calculate the likelihood weights in the same way as before, but adjust the weights by the ratio of the prior density to the updated sampling density.
6. Form a new multivariate normal sampling distribution based on the weights in the previous step, repeating steps 4 and 5 until such time as there is an acceptable degree of heterogeneity in the sample weights.
7. Draw a sample of 1 000 parameter combinations (with replacement) from the sets of parameter combinations generated in all previous steps, using the weights calculated in the last step as the sample weights. If the heterogeneity in the weights is adequate, the fraction of unique parameter combinations in the sample should be greater than $1-\exp\left(-1\right)=0.63$.

The set of 1 000 parameter combinations is effectively a sample from the posterior distribution, since sampling from the prior distribution and then resampling from this distribution using the likelihood values as sample weights is equivalent to sampling from the product of the prior distribution and likelihood function. A more detailed description of the method is provided by Raftery and Bao . The distributions of model estimates shown in the results are the distributions of results obtained when the model is run for all 1 000 parameter combinations, with the 95% confidence intervals representing the 2.5 and 97.5 percentiles of these distributions.

## 3.4 Model selection

Additional analyses were performed to assess whether it was possible to obtain a better or similar fit to the data with different assumptions. Model 1 was the same as the model described in the main text, except that it assumed no change in HIV virulence over time (i.e. *E* = 1). Model 2 was the same as Model 1, but also assumed no IeDEA-SA bias (*I_d_* = 1 for all *d*) and no dependence of mortality in the CD4 <200 category on previous ART initiation rates (*m* = 0). Model 3 was the same as the model described in the main text, except that it assumed no difference in completeness of vital registration by age and sex (i.e. using the ‘initial completeness assumptions’ in Table 7).

# 4. Additional results

## 4.1 Model calibration and validation

Fig D shows the model fit to the recorded death data by age and sex, using the main model described in the main text. (Recorded deaths have been adjusted based on the assumed levels of completeness described previously.) In general, the model fits the data closely. However, in men aged 55-59 the model under-estimates the recorded level of mortality in almost all years; this may be due to problems with the non-HIV mortality assumptions, since the model under-estimates mortality most substantially in 1997-8, when HIV-related deaths were a relatively small proportion of the total. The model also over-estimates recorded deaths in women aged 40-49 in the 2004-2007 period. In 2013 and 2014, the model over-estimates the numbers of deaths in men aged 30-39.

Fig D: Trends in modelled and recorded deaths in adults

Recorded deaths have been adjusted based on the assumed levels of completeness described in section 3.2.1.

Fig E compares the model estimates of HIV prevalence in pregnant women with those obtained in nationally-representative antenatal clinic surveys. The model estimates are again reasonably consistent with the data, although in the 20-24 age group the model tends to over-estimate HIV prevalence slightly.

Fig E: HIV prevalence in pregnant women, by age

Dots represent levels of HIV prevalence measured in antenatal surveys (95% confidence intervals are not shown for the surveys conducted prior to 1997 as the published confidence intervals for these surveys did not reflect the clustering in the survey design). Solid lines represent the posterior averages, after adjusting for antenatal bias.

Fig F compares the model estimates of HIV prevalence in the general population with those obtained in national household surveys (conducted in 2005, 2008 and 2012). Model estimates of HIV prevalence in women are very consistent with survey measurements, except in the case of women aged 15-19 in 2005, where the model under-estimates prevalence significantly. There is less consistency in the case of men, although inconsistencies by age are not sustained across successive surveys. For example, although the model estimate of HIV prevalence in men aged 55-59 in 2012 is significantly greater than that observed in the survey, the model estimates for the same age group in 2005 and 2008 are very consistent with the survey estimates.

Fig F: HIV prevalence in the general population

Dots represent prevalence levels measured in national household surveys conducted by the Human Sciences Research Council. Solid lines represent posterior averages and dashed lines represent the 2.5 and 97.5 percentiles from the posterior distributions.

Fig G shows the model fit to the recorded death data in Model 1, i.e. when assuming no change in HIV virulence over time. The model fit to the data is similar to that in the main analysis (Fig D). The model fit to the recorded death data is also similar if in addition it is assumed that there is no bias in the IeDEA-SA data and no effect of previous rates of ART initiation on mortality rates in treated and untreated individuals (Model 2, Fig H). Finally, in Model 3 (Fig I) we consider the effect of changing the completeness of vital registration assumptions to be more consistent with those in the National Burden of Disease study. The model fit to the re-adjusted data was again similar to that in Fig D.

Fig G: Trends in modelled and recorded deaths in adults when assuming no change in HIV virulence over time

Fig H: Trends in modelled and recorded deaths in adults when assuming no change in HIV virulence over time, no IeDEA-SA bias and no effect of previous ART initiation rates on mortality rates in treated and untreated individuals

Fig I: Trends in modelled and recorded deaths in adults when assuming completeness levels consistent with the Second National Burden of Disease study

Table 8 summarizes the goodness of fits statistics. Although the results in Model 1 (Fig G) are visually similar to those in the main model (Fig D), the standard deviation of the model error terms are slightly higher in Model 1 than in the main model, suggesting a slightly poorer fit to the recorded death data. In addition, the fit to the HIV prevalence data in Model 1 is not as good as that in the main analysis (*σ_u_* is 0.0058 in Model 1 compared to 0.0049 in the main analysis). Similarly, in Model 2 it is possible to obtain a fit to the mortality data that is only slightly poorer than that in the main analysis, but at the expense of the model fit to the HIV prevalence data. In Model 2, the model estimate of the total number of HIV infections in South Africa in 2012 is 5.67 million (95% CI: 5.54-5.80 million); this compares with 6.29 million (95% CI: 6.10-6.48 million) in the main analysis and 6.42 million in the 2012 HSRC household survey . In Model 3, the model fit to the recorded death data is poorer than that in the Model 1, but the fit to the HIV prevalence data is slightly better. In all of Models 1-3, the Bayes factor (on 2 times the natural log scale) is greater than 10 when comparing the model fit in the main scenario to that in the alternative analysis. Bayes factors of more than 10 (on 2 times the natural log scale) are considered ‘very strong’ evidence in favour of the comparator model , and thus the HIV prevalence and mortality data in South Africa provide very strong evidence in support of the main model over the alternative models.

Table 8: Goodness of fit statistics

| Scenario | Log of  integrated  likelihood | Bayes factor  comparing  main model to alternative model* | Standard deviation  of model errors in  fitting mortality  data (*σ_d_* in  equation 16) | Standard deviation  of model errors in  fitting HIV  prevalence data  (*σ_u_* in equation 20) |
| --- | --- | --- | --- | --- |
| Main model | 409.9 | - | 0.004399 | 0.004910 |
| Model 1 | 397.4 | 25.0 | 0.004786 | 0.005783 |
| Model 2 | 378.9 | 62.0 | 0.005014 | 0.007081 |
| Model 3 | 377.7 | 64.4 | 0.005526 | 0.004837 |

* On 2 times natural logarithm scale.

Fig J shows modelled changes in CD4 distributions in HIV-positive adults, compared with results from surveys of HIV-positive adults in different South Africa settings. The data suggest a steep decline in the fraction of HIV-positive adults with CD4 counts of less than 200 cells/μl after 2005, and this pattern is more closely matched by the model in the main analysis than in Models 1 and 2 (panel a). For the other CD4 categories, the model consistency with the data is similar across models.

Fig J: Fraction of HIV-positive adults in different CD4 categories

Dots represent data from surveys . Solid lines represent posterior averages estimated by the different models. Results are not shown for Model 2, as these are almost indistinguishable from the results for Model 1. Similarly, results are not shown for Model 3, as these are almost indistinguishable from the results in the main analysis.

Table 9 compares the posterior estimates of the model parameters in the different models. In Model 1, posterior estimates of the IeDEA-SA bias during the first 6 months after ART initiation are very different from those in the main analysis (mean of 0.57 in comparison to 1.37 in the main analysis). In Model 3, the posterior estimates are very similar to those in the main analysis, except that the difference in HIV mortality between men and women is reduced (due to the assumption that the completeness of death reporting is the same in men and women).

Table 9: Comparison of posterior distributions in alternative analyses

| Parameter | Posterior estimates (mean, 95% confidence interval) | | |
| --- | --- | --- | --- |
|  | Model 1 | Model 2 | Model 3 |
| *Untreated HIV mortality parameters* |  |  |  |
| Average male survival in absence of ART (years) | 11.49  (11.11-11.77) | 11.26  (11.31-12.06) | 12.22  (11.96-12.47) |
| RR of HIV disease progression in women | 0.940  (0.915-0.963) | 0.941  (0.914-0.978) | 0.976  (0.955-0.996) |
| Increase in HIV disease progression per  10-year increase in age | 0.259  (0.237-0.281) | 0.266  (0.241-0.293) | 0.272  (0.250-0.294) |
| RR of HIV disease progression per calendar year | - | - | 0.979  (0.975-0.984) |
| *Treated HIV mortality parameters* |  |  |  |
| IeDEA-SA bias in first 6 months of ART | 0.567  (0.441-0.0.695) | - | 1.288  (1.099-1.465) |
| Ratio of IeDEA-SA bias >42 months after ART  start to bias <6 months after ART start | 0.689  (0.590-0.788) | - | 0.884  (0.691-1.024) |
| Reduction in mortality* per unit increase in rate of  ART initiation (at CD4<200) over last 3 years | 7.49  (6.16-8.95) | - | 4.95  (4.28-5.94) |
| *HIV transmission parameters* |  |  |  |
| Ratio of increase in infectivity* to increase in  disease progression,* per unit change in SPVL | - | - | 0.119  (0.101-0.137) |
| Initial HIV prevalence in high risk women | 0.197%  (0.190-0.199%) | 0.197%  (0.188-0.200%) | 0.191%  (0.178-0.196%) |
| Male-to-female transmission probability in short-  term relationships | 0.0190  (0.0183-0.0199) | 0.0185  (0.0176-0.0194) | 0.0195  (0.0186-0.0205) |
| Female-to-male transmission probability in short-  term relationships | 0.0075  (0.0072-0.0078) | 0.0075  (0.0071-0.0079) | 0.0077  (0.0074-0.0081) |
| Reduction in unprotected sex after HIV diagnosis | 0.678  (0.606-0.745) | 0.794  (0.718-0.861) | - |

* On a natural log scale. IeDEA-SA = International Epidemiology Databases for the Evaluation of AIDS Southern Africa. RR = relative rate. SPVL = set point viral load.

Table 10 compares key model outputs in the different models. Although there is remarkable consistency in estimates of total HIV-related mortality, Model 1 yields very different estimates of the fraction of HIV deaths occurring at different levels of engagement in HIV care. In Model 1, the fraction of deaths occurring in treated individuals is much smaller than that in the main analysis, due to the lower estimated ART mortality rates in these scenarios (compare the IeDEA-SA bias parameters estimated in Table 9 with those in Table 1 of the main text). In Models 1 and 2, the cumulative number of life years saved by ART is greater than estimated in the main analysis, although the percentage reduction in HIV deaths in 2014 due to ART is estimated to be similar across scenarios. The greater number of life years saved is largely due to the estimated greater effectiveness of ART (especially in Model 1). Finally, the estimated total number of HIV infections in 2012 is roughly consistent in the main analysis and Model 3 – and also consistent with the 2012 household survey estimate of 6.4 million HIV infections . As noted previously, the estimate in Model 2 (5.67 million) is substantially lower than the other estimates, which suggests that this Model is less plausible.

Table 10: Comparison of key model outputs across scenarios

| Model output | Main  analysis | Model 1 | Model 2 | Model 3 |
| --- | --- | --- | --- | --- |
| Total adult HIV deaths up to end of 2014  (millions) | 2.70  (2.66-2.75) | 2.69  (2.65-2.74) | 2.70  (2.66-2.75) | 2.75  (2.70-2.79) |
| Adult HIV deaths in 2006 (thousands) | 231  (227-235) | 233  (229-238) | 231  (228-236) | 234  (230-239) |
| Adult HIV deaths in 2014 (thousands) | 95  (91-99) | 97  (93-102) | 102  (97-106) | 100  (96-105) |
| % of adult HIV deaths in 2014 undiagnosed | 18.3%  (17.2-19.5%) | 19.8%  (18.9-20.8%) | 17.7%  (17.0-18.4%) | 17.7%  (17.0-18.7%) |
| % of adult HIV deaths in 2014 diagnosed,  untreated | 41.7%  (34.6-48.0%) | 60.8%  (57.0-64.5%) | 45.1%  (43.1-47.0%) | 44.2%  (40.0-49.5%) |
| % of adult HIV deaths in 2014 occurring in  1^st^ 6 months after ART initiation | 10.2%  (8.6-11.9%) | 5.1%  (3.9-6.1%) | 8.2%  (7.9-8.5%) | 9.4%  (8.3-10.4%) |
| % of adult HIV deaths in 2014 occurring >6  months after ART initiation | 29.8%  (24.6-36.2%) | 14.3%  (11.3-17.6%) | 29.0%  (27.8-30.3%) | 28.7%  (24.0-32.1%) |
| Life years saved by ART by end of 2014  (millions) | 6.15  (5.52-6.69) | 8.14  (7.78-8.50) | 7.55  (7.47-7.63) | 6.29  (5.96-6.82) |
| % reduction in adult HIV deaths due to ART  in 2014 | 74.7%  (73.3-76.1%) | 77.4%  (76.4-78.2%) | 74.9%  (74.2-75.6%) | 73.9%  (72.9-75.2%) |
| Total HIV infections in 2012 (millions) | 6.29  (6.10-6.48) | 6.13  (5.97-6.30) | 5.67  (5.54-5.80) | 6.42  (6.25-6.59) |

## 4.2 Comparison of model results when fitting only to prevalence data and only to mortality data

Additional analyses were performed to assess the sensitivity of the model results to changes in the calibration data sets, and to assess the extent of conflict between calibration data sources. The model was re-calibrated using only the HIV prevalence data in the specification of the likelihood (following the approach adopted in most other HIV models that have been fitted to data from developing countries ). In addition, the model was re-calibrated using only the mortality data in the specification of the likelihood (following the approach in a few previous studies ). Conflict diagnostics were calculated for each of the model parameters, to assess the extent of the difference between parameter values when the model was fitted only to the HIV prevalence data and when the model was fitted only to the mortality data (small p values indicate strong evidence of conflict between the calibration data sets ).

Fig K compares the posterior distributions of the model parameters when the two data sources are considered separately, and shows the two-sided conflict p-values. The only parameter for which there is a highly significant difference is the relative rate of disease progression per calendar year, which represents the extent of the viral evolution. When the model is calibrated to the mortality data there is substantially stronger evidence of a change over time in the rate of disease progression than when the model is calibrated only to the HIV prevalence data. There is weak evidence of a difference in the female-to-male transmission probability (panel c), which is probably because the model estimates a lower HIV prevalence when it is calibrated only to mortality data. There is also weak evidence of a difference in the effect of age on the rate of disease progression (panel g).

Fig K: Comparison of posterior distributions for each model parameter when the model is fitted only to the HIV prevalence data and when the model is fitted only to the mortality data

Conflict p-values in each panel represent the significance of the difference in posterior distributions . Posterior distributions are calculated using kernel density estimation, with the bandwidth parameter chosen according to Silverman’s rule of thumb: 0.00002 in panel (a), 0.0003 in panel (b), 0.00013 in panel (c), 0.13 in panel (d), 0.0078 in panel I, 0.086 in panel (f), 0.0078 in panel (g), 0.55 in panel (h), 0.00019 in panel (i), 0.016 in panel (j) and 0.065 in panel (k).

Fig L compares the model estimates of adult mortality in the main analysis to those obtained when the model is fitted only to the HIV prevalence data or only to the recorded death data. Although the model estimates of adult mortality are virtually indistinguishable when comparing the model in the main analysis and the model that is fitted only to the mortality data, the model estimates of mortality are substantially higher when the model is fitted only to HIV prevalence data, particularly after 2003. The analysis that considers only the HIV prevalence data yields estimates of adult mortality substantially higher than those observed.

Fig L: Modelled and recorded trends in adult mortality (ages 20-59)

Fig M compares the model estimates of HIV prevalence in the main analysis to those obtained when the model is fitted only to the HIV prevalence data or only to the recorded death data. If the model is fitted only to the HIV prevalence data, this leads to a slightly higher estimate of adult HIV prevalence than in the main analysis, though the difference is small relative to the confidence intervals around the household survey prevalence estimates. In contrast, the model that is fitted only to the mortality data yields estimates of HIV prevalence substantially lower than those produced in the main analysis, and substantially lower than those observed in national household surveys. Thus neither data set, by itself, is sufficient to yield both realistic model estimates of trends in prevalence and realistic model estimates of trends in mortality.

Fig M: Modelled and observed changes in HIV prevalence in adults (ages 15-49)

Table 11 compares model estimates of key indicators in the three analyses. When the model is fitted only to the HIV prevalence data, estimates of cumulative AIDS mortality are 17% higher than in the main analysis, and estimates of life years saved are also substantially greater than in the main analysis (though this difference is not statistically significant). However, estimates of the total number of HIV infections in South Africa are similar to those obtained in the main analysis. In contrast, when the model is fitted only to mortality data, estimates of HIV prevalence are significantly lower than in the main analysis, but estimates of cumulative AIDS mortality and life years saved by ART are almost identical to those in the main analysis.

Table 11: Mortality and HIV prevalence outputs

| Model output | Main  analysis | Fitting only to HIV  prevalence data | Fitting only to  mortality data |
| --- | --- | --- | --- |
| Total adult HIV deaths up to end of 2014  (millions) | 2.70  (2.66-2.75) | 3.16  (2.83-3.55) | 2.68  (2.64-2.73) |
| Adult HIV deaths in 2014 (thousands) | 95  (91-99) | 161  (138-186) | 88  (84-93) |
| Life years saved by ART by end of 2014  (millions) | 6.15  (5.52-6.69) | 6.71  (5.56-8.02) | 6.16  (5.73-6.65) |
| Reduction in HIV deaths by end of 2014  (millions), as a result of ART | 1.72  (1.58-1.84) | 1.85  (1.64-2.07) | 1.70  (1.61-1.80) |
| Total HIV infections in 2012 (millions) | 6.29  (6.10-6.48) | 6.44  (6.21-6.68) | 4.98  (4.78-5.28) |

## 4.3 Comparison with recently-published AIDS mortality estimates from other models

Fig N compares the estimates of numbers of HIV-related deaths in South Africa produced by different agencies/models. (For the purpose of this comparison we have added the Thembisa model estimates of HIV-related deaths in adults to the model estimates of HIV-related deaths in children; a more detailed description of the Thembisa modelling of HIV in children is provided elsewhere ). The Thembisa estimates of mortality are roughly consistent with the results of the South African National Burden of Disease study , but substantially lower than the estimates published by UNAIDS and the IHME .

Fig N: Deaths attributable to HIV in South Africa

# References

# Appendix A: Modelling of changes in mortality at low CD4 counts

To describe pre-ART mortality at CD4 counts below 200/μl, we assume that the untreated HIV mortality rate in individuals with CD4 count *x* is

$\mu(x)=a\left(b^{x}\right)$, (A1)

where *a* is the mortality rate we would expect in an untreated individual with a CD4 count of zero, and *b* is the factor by which the mortality rate decreases per unit increase in the CD4 count. The *b* parameter is estimated by fitting regression models of the form given in equation (A1) to average mortality levels reported over different CD4 ranges, in different African studies conducted prior to the availability of ART . The resulting model fits to the data are shown in Fig A1. Although the overall mortality levels in the Malawian setting are higher than those in Uganda , Côte d’Ivoire and South Africa , the fitted *b* parameter values are remarkably consistent across settings: 0.9882 in Malawi, 0.9876 in Uganda, 0.9899 in Côte d’Ivoire and 0.9887 in South Africa. We therefore set the *b* parameter in our model at the average of these values, 0.9886.

Fig A1: Effect of CD4 count on mortality in the absence of ART

For the purpose of fitting the models to the data points, the average mortality rates reported over different ranges have been taken to apply at the midpoints of the relevant ranges. Mortality data from the 200-350 CD4 range have been included in order to increase the statistical confidence in the fitted parameters.

The *a* parameter has been set to produce estimates consistent with previously-estimated South African mortality rates in the CD4 200-349 and <200 categories, 0.027 and 0.21 respectively . If 0.027 is the average mortality rate that would be expected at a CD4 count of 275/μl, then substituting these values into equation (A1) together with the previously-estimated value of *b* gives us *a* = 0.645. (As a validity check, the implied mortality at a CD4 count of 100 is 0.645 × 0.9886^100^ = 0.205, which is close to the average of 0.21 that we would expect over the CD4 <200 range.)

Suppose that *q_t_* is the annual mortality rate in untreated adults with CD4 counts <200, in year *t*. Further suppose that *q*_0_ is the corresponding mortality rate that would have been expected in the absence of any ART rollout, and that *q*_min_ is the minimum mortality rate that we would expect if rates of ART initiation were at their maximum. We would expect *q_t_* to decline as the rate of ART initiation increases, as high rates of ART initiation imply that few individuals will progress to very low CD4 counts (<50) without starting ART. In modelling *q_t_* we assume it is exponentially related to $r_{t-}$, the average rate of ART initiation over the previous three years, subject to the maximum of *q*_0_ and the minimum of *q*_min_:

$q_{t}=q_{\min}+\left(q_{0}-q_{\min}\right)\exp\left(-mr_{t-}\right)$, (A2)

where *m* is the assumed exponential parameter. This can be written as

$A_{t}=\frac{q_{\min}}{q_{0}}+\left(1-\frac{q_{\min}}{q_{0}}\right)\exp\left(-mr_{t-}\right)$, (A3)

where $A_{t}\equiv q_{t}/q_{0}$is an adjustment factor applied to the mortality rate that would be expected in the absence of any ART rollout. The ratio $q_{\min}/q_{0}$ can be estimated by noting that untreated mortality is at a minimum when all patients start ART soon after their CD4 count drops below 200. Setting *q*_min_ = *μ*(200) = 0.065 and setting *q*_0_ = 0.21 yields $q_{\min}/q_{0}$= 0.31. The *m* parameter is difficult to quantify precisely, so a Bayesian approach is adopted to reflect the uncertainty regarding this parameter (discussed further below).

A similar approach is adopted in modelling mortality during the first 6 months after starting ART. Suppose that *v_t_* is the annual mortality rate in adults during their first 6 months after starting ART (with baseline CD4 counts <200), in year *t*. Further suppose that *v*_0_ is the corresponding mortality rate that would have been expected in the very early stages of the ART rollout, when rates of ART initiation were very low, and that *v*_min_ is the minimum mortality rate that we would expect if rates of ART initiation were at their maximum. We would expect *v_t_* to decrease as the rate of ART rollout increases, as higher rates of ART rollout should lead to higher baseline CD4 counts. As before, we assume a relationship of the form

$B_{t}=\frac{v_{\min}}{v_{0}}+\left(1-\frac{v_{\min}}{v_{0}}\right)\exp\left(-mr_{t-}\right)$, (A4)

where $B_{t}\equiv v_{t}/v_{0}$. Note that the *m* parameter is assumed to be the same as that in equation (A2), although one could argue that the relationship with the rate of ART initiation may differ depending on whether one is considering pre-ART mortality or treated mortality. (In the interests of obtaining a parsimonious model fit, we use the same parameter value in equations (A2) and (A4), but the model does allow for different values to be assumed.)

For the purpose of estimating the ratio $v_{\min}/v_{0}$, we will assume that the mortality rate for individuals with baseline CD4 counts of *x*, *v*(*x*), is of the form

$v(x)=z\left(h^{x}\right)$. (A5)

Studies suggest that in the early stages of South Africa’s ART rollout, baseline CD4 distributions were roughly uniform over the range (0, 200) , which suggests that we can approximate *v*_0_ using the formula

$v_{0}=\int_{0}^{200} \frac{1}{200}z\left(h^{x}\right) dx=\frac{z}{200\ln(h)}\left(h^{200}-1\right)$. (A6)

Since the minimum mortality rate in the first 6 months of ART would be achieved if all patients starting ART at CD4 <200 cells/μl had initial CD4 counts close to 200, $v_{\min}=v(200)=z\left(h^{200}\right)$. From this it follows that

$\frac{v_{\min}}{v_{0}}=\frac{200\ln(h)}{1-h^{-200}}$, (A7)

which is independent of *z*. Estimating the ratio $v_{\min}/v_{0}$ therefore requires only that we have estimates of the parameter *h*, which can be obtained from various studies that have estimated the effect of baseline CD4 count on mortality during the first 6 months of ART. Table A1 summarizes relevant studies that have been conducted in South Africa. Although most of these studies report hazard ratios for CD4 intervals, it is possible to convert these into continuous CD4 effects using simple exponential regression models if the mortality hazards are assumed to apply to the average CD4 count over each interval (as in Fig A1). Table A1 shows the estimates of the *h* parameter that have been obtained by fitting these exponential models in each case. The average value, 0.9917, is substituted into equation (A7) to obtain a $v_{\min}/v_{0}$ estimate of 0.39.

Table A1: South African studies of effect of baseline CD4 count on early ART mortality

| Study | Population/location | ART durations | Factor by which  mortality decreases  per unit increase in  baseline CD4 (*h*) |
| --- | --- | --- | --- |
| Lawn *et al* | Gugulethu programme,  Cape Town | <4 months | 0.9905 |
| Hoffmann *et al* | Aurum community and  workplace programmes | 13 weeks | 0.9928 |
| Boulle *et al* | Khayelitsha programme,  Cape Town | <3 months | 0.9923 |
| Cornell *et al* | IeDEA-SA Collaboration | <4 months | 0.9874 |
| Mutevedzi *et al* | Africa Centre, KwaZulu-  Natal, ages <50 | <3 months | 0.9943 |
| Leisegang *et al* | Aid for AIDS programme | <4 months | 0.9927 |
| Average |  |  | 0.9917 |

For the model of early ART mortality, it is possible to obtain a crude estimate of the *m* parameter by using data on early mortality from the Western Cape , together with estimates of rates of ART initiation in the province . Table A2 shows the estimated probability of death during the first 6 months after starting ART, for patients starting ART in each calendar year, as well as rates of ART initiation derived from the previously-published model of ART coverage. Fitting a simple regression model of the form given in equation (A4) to the estimates of *v_t_* and *r_t_* in Table A2 yields an estimate of the *m* parameter equal to 15.7, if *v*_0_ is set to 0.28 and the ratio $v_{\min}/v_{0}$ is set to 0.39. (The rate of 0.28 was chosen as it is close to the rate estimated in 2001, when the ART rollout in the Western Cape had just started.) It is likely that this *m* estimate is an over-estimate, as the recording of mortality in patient record systems has become less complete over time as an increasingly high proportion of patients has been classified ‘lost to follow-up’ , and in other settings where vital registration has remained relatively complete, reductions in early mortality have been more moderate . This implies that some of the apparent improvement in early ART mortality, which we are attributing to the effect of rising rates of ART initiation, is actually due to declining completeness of death reporting. A more moderate decline in mortality over time would lead to a lower estimate of *m*, and the 15.7 estimate is therefore likely to be an upper bound. To represent our uncertainty regarding the *m* parameter, we have chosen a gamma prior with a mean of 7.5 and a standard deviation of 3.5. The 2.5 and 97.5 percentiles of this distribution are 2.3 and 15.8 respectively, reflecting the substantial prior uncertainty that exists for this parameter.

Table A2: Early ART mortality and ART initiation in the Western Cape province

|  | 2000 | 2001 | 2002 | 2003 | 2004 | 2005 |
| --- | --- | --- | --- | --- | --- | --- |
| 6-month cumulative mortality^*^ | - | 0.127 | 0.117 | 0.096 | 0.060 | 0.066 |
| Annual mortality rate (*v_t_*) | - | 0.2716 | 0.2489 | 0.2019 | 0.1238 | 0.1366 |
| Adults starting ART† | 158 | 391 | 691 | 2585 | 5468 | 8528 |
| Untreated adults with CD4 <200  cells/μl or AIDS (mid-year)† | 10706 | 14286 | 18296 | 22131 | 23465 | 23440 |
| ART initiation rate (*r_t_*) | 0.0148 | 0.0274 | 0.0378 | 0.1168 | 0.2330 | 0.3638 |
| Average ART initiation rate over  previous 3 years (*r_t_*-)‡ | - | 0.0049 | 0.0211 | 0.0266 | 0.0607 | 0.1292 |

* From Boulle *et al* . † From the model described by Adam and Johnson . ‡ Assuming zero ART initiation rate prior to 2000.

Finally, we define *w_t_* to be the annual mortality rate in ART patients in year *t*, who have been on ART for durations >6 months, having started ART with an initial CD4 count <200 cells/μl. As with *v_t_*, we would expect this rate to decline with respect to *t* as rates of ART initiation increase. However, we would expect the decline in *w_t_* to be more moderate than that in *v_t_*, since mortality at longer ART durations is not as strongly related to baseline CD4 count as mortality at early ART durations. We define a relation between *w_t_* to and *r_t_*- similar to that in equation (A4):

$w_{t}=w_{\min}+\left(w_{0}-w_{\min}\right)\exp\left(-mr_{t-}\right)$, (A8)

where *w*_0_ is the mortality rate that would have been expected in the very early stages of the ART rollout, and *w*_min_ is the minimum mortality rate that we would expect if rates of ART initiation were at their maximum. As before, we define $C_{t}\equiv w_{t}/w_{0}$, so that

$C_{t}=\frac{w_{\min}}{w_{0}}+\left(1-\frac{w_{\min}}{w_{0}}\right)\exp\left(-mr_{t-}\right)$. (A9)

For the purpose of estimating the ratio $w_{\min}/w_{0}$, we use the same assumptions as before to derive a similar formula to that in equation (A7),

$\frac{w_{\min}}{w_{0}}=\frac{200\ln(f)}{1-f^{-200}}$. (A10)

where *f* is the factor by which mortality at durations >6 months reduces, per unit increase in the baseline CD4 count. Estimates of the *f* parameter are obtained in the same way as before, based on fitting simple exponential regression models to hazard ratio estimates from various published studies (Table A3). There are relatively few such studies, as most studies control for time-updated CD4 count (rather than baseline CD4 count) when assessing the effect of CD4 count on mortality at longer ART durations. Substituting the average estimate of *f*, 0.9955, into equation (A10) gives an estimate for $w_{\min}/w_{0}$ of 0.61. As expected, this is higher than the ratio of 0.39 estimated for $v_{\min}/v_{0}$, reflecting the more modest effect of baseline CD4 count on mortality at longer ART durations.

Table A3: South African studies of effect of baseline CD4 count on late ART mortality

| Study | Population/location | ART durations | Factor by which  mortality decreases  per unit increase in  baseline CD4 (*f*) |
| --- | --- | --- | --- |
| Hoffmann *et al* | Aurum community and  workplace programmes | 52 weeks | 0.9931 |
| Johnson *et al* | IeDEA-SA  Collaboration, males | >12 months | 0.9966 |
|  | IeDEA-SA  Collaboration, females | >12 months | 0.9966 |
| Cornell *et al* | IeDEA-SA  Collaboration | 12-36 months | 0.9955 |
| Average |  |  | 0.9955 |

A limitation of the approach described above is that it considers only the uncertainty in the *m* parameter. There is also uncertainty regarding the $q_{\min}/q_{0}$, $v_{\min}/v_{0}$ and $w_{\min}/w_{0}$ ratios. We consider here the extent to which the results of the Bayesian analysis change if we allow for uncertainty in the $q_{\min}/q_{0}$ ratio in the model fitting procedure. (For the purpose of this analysis, we do not consider the uncertainty in the $v_{\min}/v_{0}$ and $w_{\min}/w_{0}$ ratios, as the results in Fig 3 of the main text show that almost all HIV-related deaths occur in untreated individuals, so uncertainty regarding the maximum possible improvement in the mortality rates of treated patients with baseline CD4 counts <200 cells/µl is unlikely to change the conclusions substantially. In addition, the IMIS posterior simulation did not converge if these additional parameters were allowed to vary in the model fitting procedure, and it was therefore necessary to limit the uncertainty analysis to the parameters that were considered most influential.)

Firstly, we specify a prior distribution on the $q_{\min}/q_{0}$ ratio. As noted previously, this ratio is approximately equal to $\mu(200)/\mu(100)=b^{100}$. Using the delta method, we can thus express the uncertainty in the $q_{\min}/q_{0}$ ratio in terms of the uncertainty in *b*. The standard deviation of the *b* values estimated previously is 0.000976, and the corresponding standard deviation in $q_{\min}/q_{0}$ is 0.0314. We thus assign a beta prior to represent the uncertainty in this ratio, with a mean of 0.31 (the same as the value assumed in the initial analysis) and a standard deviation of 0.0314. The model is fitted to the HIV prevalence data and mortality data in the same way as before.

Table A4 compares the posterior estimates of the model parameters in this alternative analysis to the posterior estimates in the main analysis. Posterior distributions are mostly very similar, although the estimates of the IeDEA bias parameters are slightly closer to 1. Importantly, the posterior mean of the $q_{\min}/q_{0}$ ratio is 0.315, very similar to the prior mean. Allowing for uncertainty in the$q_{\min}/q_{0}$ ratio therefore does not lead to substantial differences in the posterior estimates of the key model parameters.

Table A4: Comparison of posterior distributions in alternative analyses

| Parameter | Posterior estimates (mean, 95% confidence interval) | | |
| --- | --- | --- | --- |
|  | Main  analysis | Allowing for  uncertainty in  *q_min_*/q_0_ ratio |  |
| *Untreated HIV mortality parameters* |  |  |  |
| Average male survival in absence of ART (years) | 11.48 (11.17-11.82) | 11.47 (11.21-11.67) |  |
| RR of HIV disease progression in women | 0.921 (0.896-0.948) | 0.924 (0.908-0.939) |  |
| Increase in HIV disease progression per  10-year increase in age | 0.235 (0.209-0.259) | 0.239 (0.221-0.258) |  |
| RR of HIV disease progression per calendar year | 0.978 (0.972-0.983) | 0.978 (0.977-0.980) |  |
| *Treated HIV mortality parameters* |  |  |  |
| IeDEA-SA bias in first 6 months of ART | 1.368 (1.068-1.708) | 1.271 (1.187-1.378) |  |
| Ratio of IeDEA-SA bias >42 months after ART  start to bias <6 months after ART start | 0.619 (0.525-0.706) | 0.734 (0.715-0.756) |  |
| Reduction in mortality* per unit increase in rate of  ART initiation (at CD4<200) over last 3 years | 4.76 (3.71-5.75) | 4.75 (4.12-5.47) |  |
| Ratio of minimum mortality in untreated CD4<200  group to baseline mortality in untreated CD4<200  group (pre-ART era) | 0.31† | 0.315 (0.303-0.328) |  |
| *HIV transmission parameters* |  |  |  |
| Ratio of increase in infectivity* to increase in  disease progression,* per unit change in SPVL | 0.111 (0.095-0.128) | 0.114 (0.103-0.127) |  |
| Initial HIV prevalence in high risk women | 0.193% (0.184-0.198%) | 0.189% (0.179-0.193%) |  |
| Male-to-female transmission probability in short-  term relationships | 0.0198 (0.0188-0.0206) | 0.0197 (0.0190-0.0204) |  |
| Female-to-male transmission probability in short-  term relationships | 0.0075 (0.0072-0.0079) | 0.0075 (0.0072-0.0078) |  |

* On a natural log scale. † Parameter not included in the main uncertainty analysis. IeDEA-SA = International Epidemiology Databases for the Evaluation of AIDS Southern Africa. RR = relative rate. SPVL = set point viral load.

Similarly, the inclusion of the uncertainty in the $q_{\min}/q_{0}$ ratio in the model fitting procedure does not lead to substantial differences in the estimates of key model outputs (Table A5). The Bayes factor comparing the integrated likelihood in the main analysis to that when allowing for uncertainty in the $q_{\min}/q_{0}$ ratio is 5.89 on the 2 × natural log scale, which is not considered strong evidence of a superior model fit in the main analysis .

Table A5: Comparison of key model outputs in alternative analyses

| Model output | Main  analysis | Allowing for  uncertainty in  *q_min_*/q_0_ ratio |
| --- | --- | --- |
| Total adult HIV deaths up to end of 2014 (millions) | 2.70 (2.66-2.75) | 2.70 (2.66-2.74) |
| Adult HIV deaths in 2006 (thousands) | 231 (227-235) | 231 (227-234) |
| Adult HIV deaths in 2014 (thousands) | 95 (91-99) | 96 (92-99) |
| Total HIV infections in 2000 (millions) | 3.14 (3.03-3.27) | 3.13 (3.02-3.22) |
| Total HIV infections in 2012 (millions) | 6.29 (6.10-6.48) | 6.27 (6.13-6.43) |

1. In some cases we use the prior means rather than the posterior means, as the prior means were in these instances found to yield estimates of CD4 distributions more consistent with empirical data when entered into the more sophisticated Thembisa model (see Fig J). [↑](#footnote-ref-2)
